# The ADAP-TRIM21-ZEB2 axis regulates CD8^+^ T cell effector expansion and central memory-like differentiation

**DOI:** 10.1016/j.isci.2026.117016

**Published:** 2026-08-01

**Authors:** Jiajia Lu, Fan Yang, Yanli Li, Pengchao Zhang, Tengfei Jia, Hebin Liu

**Affiliations:** 1 Institutes of Biology and Medical Sciences (IBMS), Soochow University, Suzhou, Jiangsu, China; 2 Drug Discovery, Faculty of Science and Engineering, University of Nottingham Ningbo China, Ningbo, Zhejiang, China

**Keywords:** ADAP, CD8^+^ T cell, ZEB2, memory, effector function

## Abstract

The ADAP (adhesion and degranulation-promoting adapter protein)-SKAP1 signaling module is essential for TCR-mediated activation and LFA-1-dependent adhesion; however, its role in coordinating CD8^+^ T cell differentiation remains unclear. Here, we show that ADAP acts as a negative regulator of CD8^+^ T cell response. During acute LCMV-Armstrong infection, ADAP deficiency enhances CD8^+^ T cell effector expansion and function, as evidenced by increased IFN-γ and granzyme B expression, and subsequently favors the formation of central memory-like CD8^+^ T cells (CD44^Hi^ CD62L^Hi^). RNA sequencing (RNA-seq) analysis identified ZEB2 as one of the most significantly upregulated transcription factors in *Adap*^−/−^ CD8^+^ T cells. Mechanistically, ADAP interacts with the E3 ubiquitin ligase TRIM21 to facilitate K48-linked polyubiquitination and degradation of ZEB2. This, in turn, reduces the binding of ZEB2 to the *Smad3* promoter. Collectively, our study identifies a previously uncharacterized ADAP-TRIM21-ZEB2 axis that regulates CD8^+^ T cell differentiation, providing new mechanistic insights into the control of T cell fate decisions.

## Highlights

• ADAP deficiency enhances effector expansion and cytotoxic function of CD8^+^ T cells
• Loss of ADAP promotes central memory-like (CD44^Hi^ CD62L^Hi^) CD8^+^ T cell formation
• ZEB2 is a key upregulated transcription factor in *Adap*^−/−^ CD8^+^ T cells
• ADAP recruits TRIM21 to mediate ZEB2 ubiquitination and control CD8^+^ T cell fate

## Graphical abstract

**Figure:**
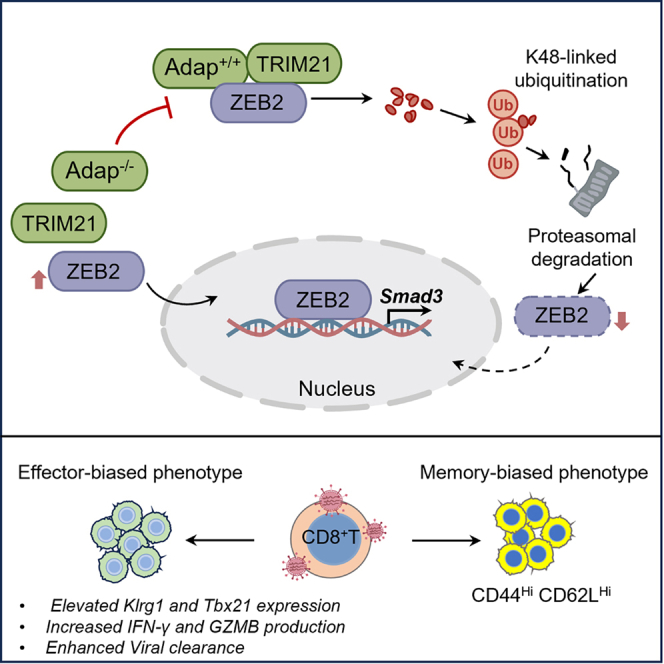

## Teaser

molecular biology; immunology; cell biology

## Introduction

CD8^+^ T cells are pivotal mediators of adaptive immunity, providing defense against viral infections and malignancies through effector responses and long-term memory formation.^1^ Upon encountering antigens, naive CD8^+^ T cells undergo activation, clonal expansion, and differentiation into effector T cells (T_EFF_), which secrete IFN-γ, TNF-α, and granzymes.^2^^,^^3^^,^^4^^,^^5^ This effector pool is heterogeneous, comprising short-lived effector cells (SLECs; KLRG1^+^ CD127^-^) and memory precursor effector cells (MPECs; KLRG1^-^ CD127^+^), the latter of which give rise to long-lived memory subsets.^3^^,^^6^^,^^7^ Memory CD8^+^ T cells are broadly categorized as central (T_CM_), effector (T_EM_), and tissue-resident (T_RM_) cells, each with distinct migratory and functional properties.^8^^,^^9^^,^^10^ While T_EM_ and T_RM_ cells localize to peripheral tissues for rapid pathogen control, T_CM_ cells circulate through lymphoid organs, maintaining long-term immunity via self-renewal and recall capacity.^11^^,^^12^^,^^13^^,^^14^^,^^15^ Typically, only 5%–10% of T_EFF_ cells survive antigen clearance to establish durable memory that enables rapid recall responses upon re-exposure.^16^ However, under conditions of persistent antigen stimulation, such as cancer or chronic infections, CD8^+^ T cells progressively differentiate toward a dysfunctional state of exhaustion.^17^ Accumulating evidence indicates that antigenic properties, dose, affinity, and duration are critical determinants in shaping CD8^+^ T cell fate decisions.^18^^,^^19^^,^^20^^,^^21^^,^^22^ Moderate TCR signaling, induced by low-dose or low-affinity antigens, favors the maintenance of stem-like and memory precursor populations, whereas strong or prolonged stimulation drives terminal effector differentiation or exhaustion.^23^^,^^24^ Moreover, the physicochemical characteristics of the TCR-pMHC interface, including hydrophobicity and polarity, can fine-tune signal strength and thereby bias CD8^+^ T cells toward effector or memory differentiation.^25^^,^^26^

The differentiation of CD8^+^ T cells into effector or memory states is tightly regulated by transcriptional networks. Multiple transcription factors that regulate the ability of CD8^+^ T cells to adopt effector or memory CD8^+^ T cell fates have been identified, many of which operate in a dynamic and graded manner, generating an intricate layered system of transcriptional states reflecting the integration of environmental signals that individual cells experience over the course of an infection.^27^^,^^28^^,^^29^ ZEB2 (also known as Zfhx1b and Sip1) promotes effector commitment by suppressing memory-associated genes and synergizing with T-bet to enforce terminal differentiation.^3^^,^^30^^,^^31^ ZEB2 activity is modulated by TGF-β/SMAD signaling and microRNA-200 family members.^32^^,^^33^^,^^34^^,^^35^ ZEB2 can also mediate transcriptional repression via cooperation with activated SMADs or through the recruitment of the corepressor CtBP as well as histone deacetylase complexes, particularly NuRD,^36^^,^^37^^,^^38^ yet the upstream regulators governing its stability during T cell differentiation remain incompletely undefined.

Tripartite motif-containing 21 (TRIM21) is an E3 ubiquitin ligase originally characterized as a cytosolic Fc receptor involved in antiviral immunity.^39^^,^^40^ Emerging evidence has implicated TRIM21 as a negative regulator of CD8^+^ T cell function, primarily through the modulation of immune checkpoint signaling and T cell activation thresholds.^41^ Notably, TRIM21 was shown to restrain CD8^+^ T cell antitumor activity by stabilizing inhibitory receptors such as PD-1 via non-degradative ubiquitination.^41^ However, whether TRIM21 directly regulates transcriptional programs governing CD8^+^ T cell effector differentiation and memory formation remains largely unexplored.

Adhesion and degranulation-promoting adapter protein (ADAP, also known as FYB or SLAP-130) was initially identified as an immune adapter protein implicated in dual-directional signaling within T cells.^42^ ADAP facilitates HIV infection in a T cell-dependent mechanism.^43^ ADAP integrates TCR signaling by coupling with SLP-76 and forms a stable complex with SKAP1 (Src kinase-associated phosphoprotein 1) to promote integrin-dependent adhesion between T cells and antigen-presenting cells.^44^^,^^45^ Interestingly, while SKAP1 expression is preserved in the absence of ADAP, ADAP deficiency destabilizes SKAP1, indicating that SKAP1 is dependent on ADAP.^46^^,^^47^ ADAP also contributes to TCR-induced NF-κB activation via interaction with CARMA1 and modulates integrin signaling through its interaction with the SUMO-conjugating enzyme Ubc9.^48^^,^^49^ Moreover, ADAP can reset macrophage function by controlling the TLR4-induced upregulation of PDPN as a host innate immune defense during sepsis.^50^ In addition to its canonical signaling role, ADAP has emerged as a regulator of CD8^+^ T cell differentiation. ADAP-deficient mice exhibit expanded populations of antigen-specific CD44^Hi^ CD122^Hi^ CD8^+^ T cells under steady-state and infection conditions, suggesting that ADAP constrains memory formation.^51^ Although ADAP is dispensable for CD8^+^ T cell effector function, its loss impairs T_RM_ cell effector molecule expression and cytokine responsiveness (e.g., IL-15).^51^^,^^52^ Collectively, these findings imply context-dependent functions of ADAP in balancing effector and memory CD8^+^ T cell differentiation, but the underlying molecular mechanisms connecting ADAP to transcriptional regulation remain to be elucidated.

Here, we demonstrate that ADAP constrains CD8^+^ T cell effector expansion and memory formation during acute viral infection. Mechanistically, ADAP recruits the E3 ubiquitin ligase TRIM21 to mediate the K48-linked ubiquitination of ZEB2, restricting its binding to the *Smad3* promoter. Our findings identify the ADAP-TRIM21-ZEB2 axis as a critical checkpoint in CD8^+^ T cell differentiation, offering insights for optimizing memory responses in antiviral infection and immunotherapies.

## Results

### ADAP deficiency promotes the expansion of effector and central memory-like CD8^+^ T cells

**Figure 1:**
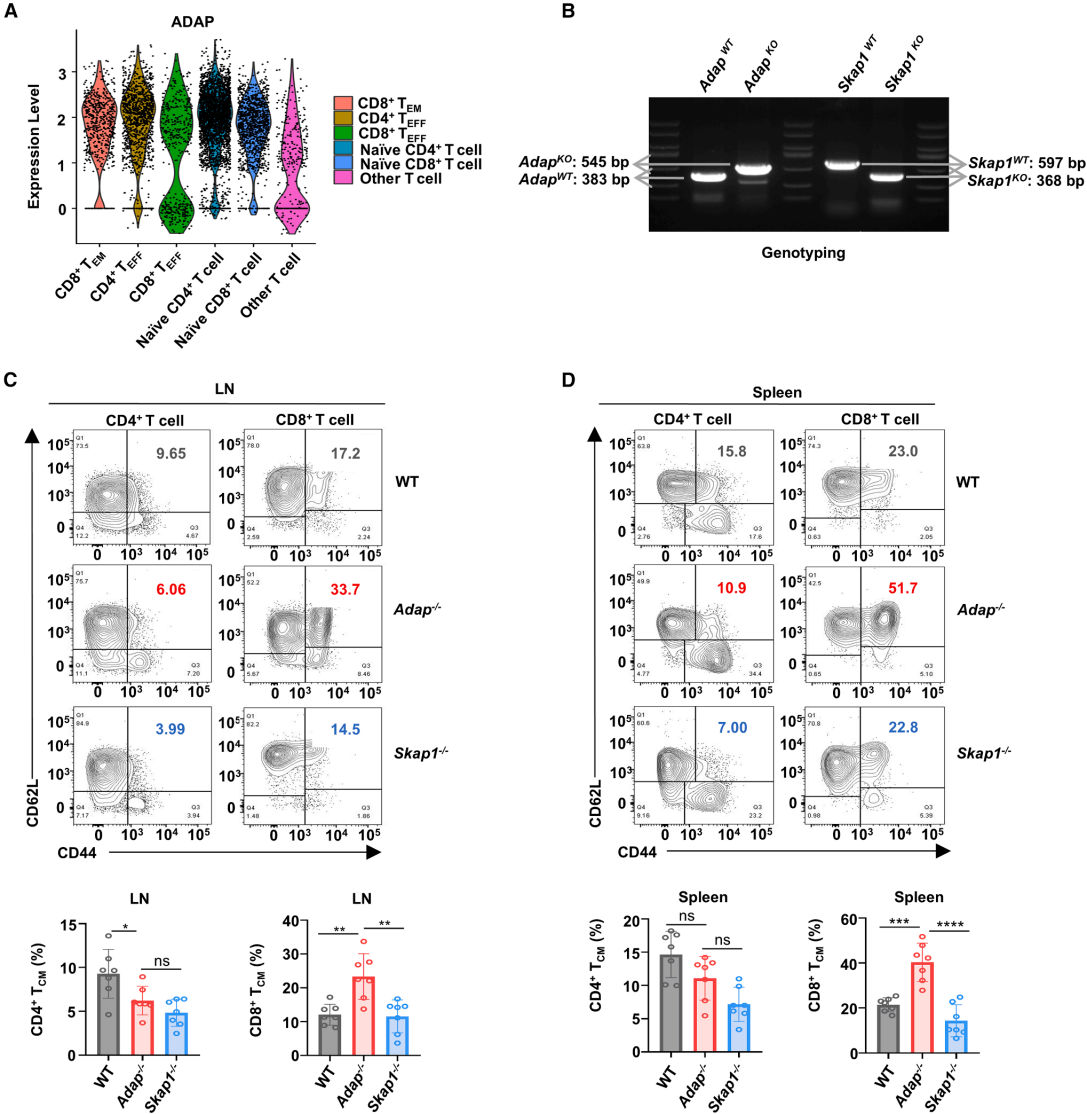
Loss of ADAP promotes the generation of a mature state of CD8^+^ T cells with increased ratio of CD8^+^ T_CM_-like T cells (A) Violin plot of ADAP expression in different T cell subsets from the GEO: GSE150861 public dataset. (B) Representative PCR amplification-mediated genotyping of *Adap*^−/−^ and *Skap1*^−/−^ mice. (*n* = 3 independent repeat experiments). (C and D) Flow cytometry analysis of CD44^Hi^ CD62L^Hi^ T_CM_-like cells gated on total CD4^+^/CD8^+^ T cells from the lymph nodes (C) (*n* = 7 mice/group, one-way ANOVA and Tukey’s multiple comparison) and the spleens (D) (*n* = 7 mice/group, one-way ANOVA and Tukey’s multiple comparison). The bar charts show the comparison of T_CM_-like cell frequencies in WT, *Adap*^−/−^, and *Skap1*^−/−^ mice. All data are expressed as mean with SD. *n* represents biological replicates per group. ∗*p* < 0.05, ∗∗*p* < 0.01, ∗∗∗*p* < 0.001, ∗∗∗∗*p* < 0.0001; ns, not significant.

To explore the relationship between ADAP expression and T cell subset dynamics during antiviral immune responses, we analyzed single-cell RNA sequencing (RNA-seq) data from the NCBI public database (GEO: GSE150861), comprising peripheral blood mononuclear cell (PBMC) samples from healthy donors and COVID-19 patients. Violin plot visualization showed the general expression of ADAP in different T cell subsets (Figure 1A). To further investigate the role of ADAP in this context, we used ADAP knockout C57BL/6J mice,^53^ as confirmed by representative PCR amplification-mediated genotyping (Figure 1B). To determine which subsets of T cells are specifically affected upon *Adap* depletion, CD4^+^ and CD8^+^ T cells from the spleen and lymph nodes of Wild-type (WT), *Adap*^−/−^,and *Skap1*^−/−^ mice were gated and subjected to flow cytometry analysis with antibodies against CD44 and CD62L. Under steady-state conditions, compared with WT and *Skap1*^−/−^ mice, *Adap*^−/−^ mice presented a significantly greater frequency of a specific population of T_CM_ memory-like CD8^+^ T cells, which were characterized by high expression levels of CD44 and CD62L (CD44^Hi^ CD62L^Hi^) (Figures 1C and 1D), without a noticeable change in the overall number of CD8^+^ T cells. In contrast, no obvious differences were noted among the corresponding subsets within the CD4^+^ T cell population. These data suggest that ADAP selectively regulates the differentiation of T_CM_-like (CD44^Hi^ CD62L^Hi^) CD8^+^ T cells, but not CD4^+^ T cells.

**Figure 2:**
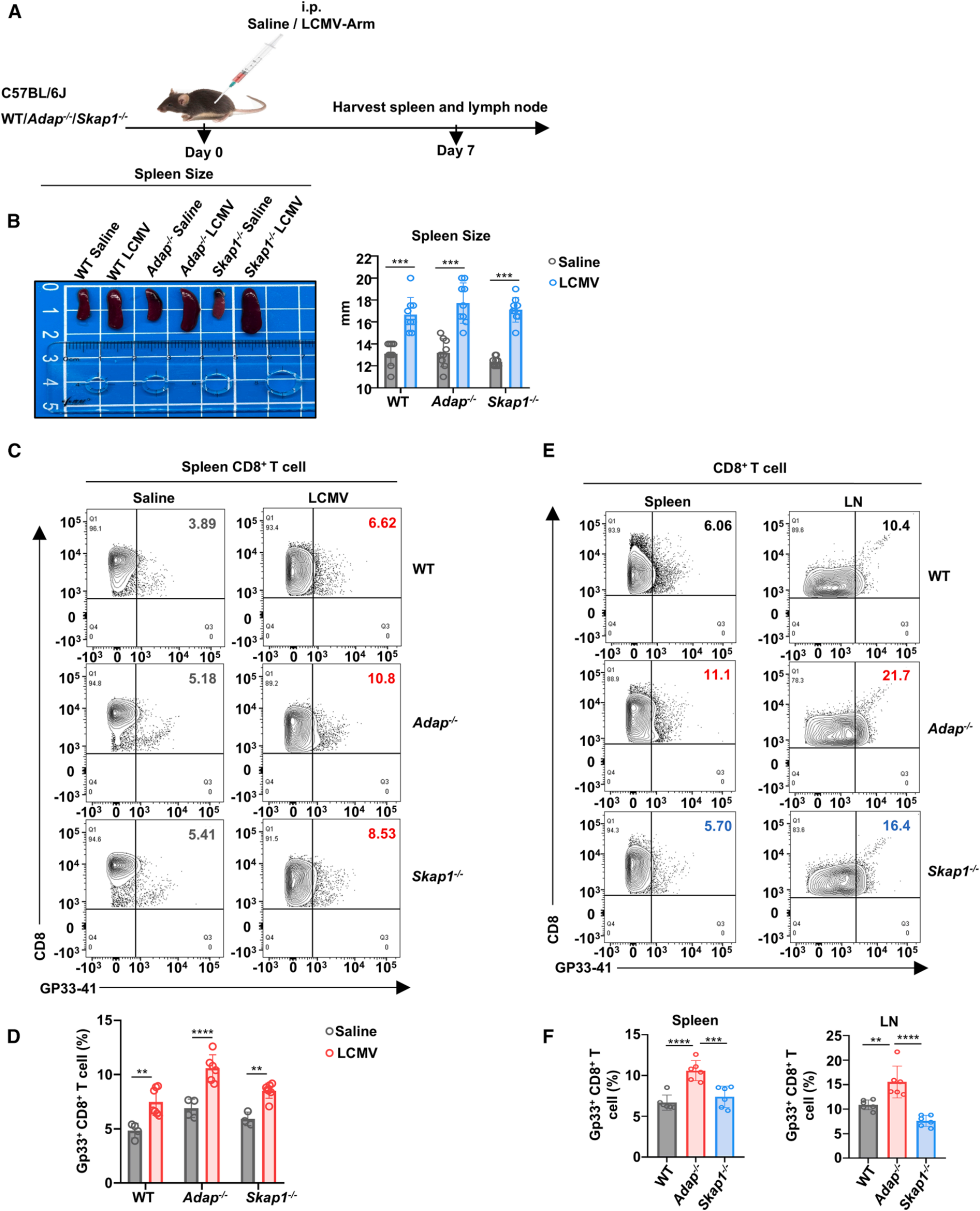
ADAP deficiency upregulated the proportion of antigen-specific CD8^+^ T cells (A) Schematic diagram of the experimental design and procedure for identifying CD8^+^ T cells in the spleens and the lymph nodes of WT, *Adap*^−/−^, and *Skap1*^−/−^ mice either uninfected or infected with LCMV-Arm. (B) On day 7 after LCMV-Arm infection, the spleens were photographed. Bar charts showing the comparison of the mean size of the spleen in WT, *Adap*^−/−^, and *Skap1*^−/−^ mice (*n* = 8 mice/group, two-way ANOVA and Tukey’s multiple comparison). (C and D) On day 7 after LCMV-Arm infection or saline control, the spleen Gp33-specific CD8^+^ T cells were harvested and subjected to flow cytometry analysis. Representative contour plots showing Gp33-specific CD8^+^ T cells from WT, *Adap*^−/−^, and *Skap1*^−/−^ mice. The frequency of the indicated subsets is shown in (D) (*n* ≥ 3 mice/group, two-way ANOVA and Tukey’s multiple comparison). (E and F) Representative contour plots showing spleen and lymph node Gp33-specific CD8^+^ T cells from WT, *Adap*^−/−^, and *Skap1*^−/−^ mice on day 7 p.i. The frequency of the indicated subsets is shown in (F) (*n* = 6 mice/group, one-way ANOVA, and Tukey’s multiple comparison). All data are expressed as mean with SD. *n* represents biological replicates per group. ∗∗*p* < 0.01, ∗∗∗*p* < 0.001, ∗∗∗∗*p* < 0.0001.

**Figure 3:**
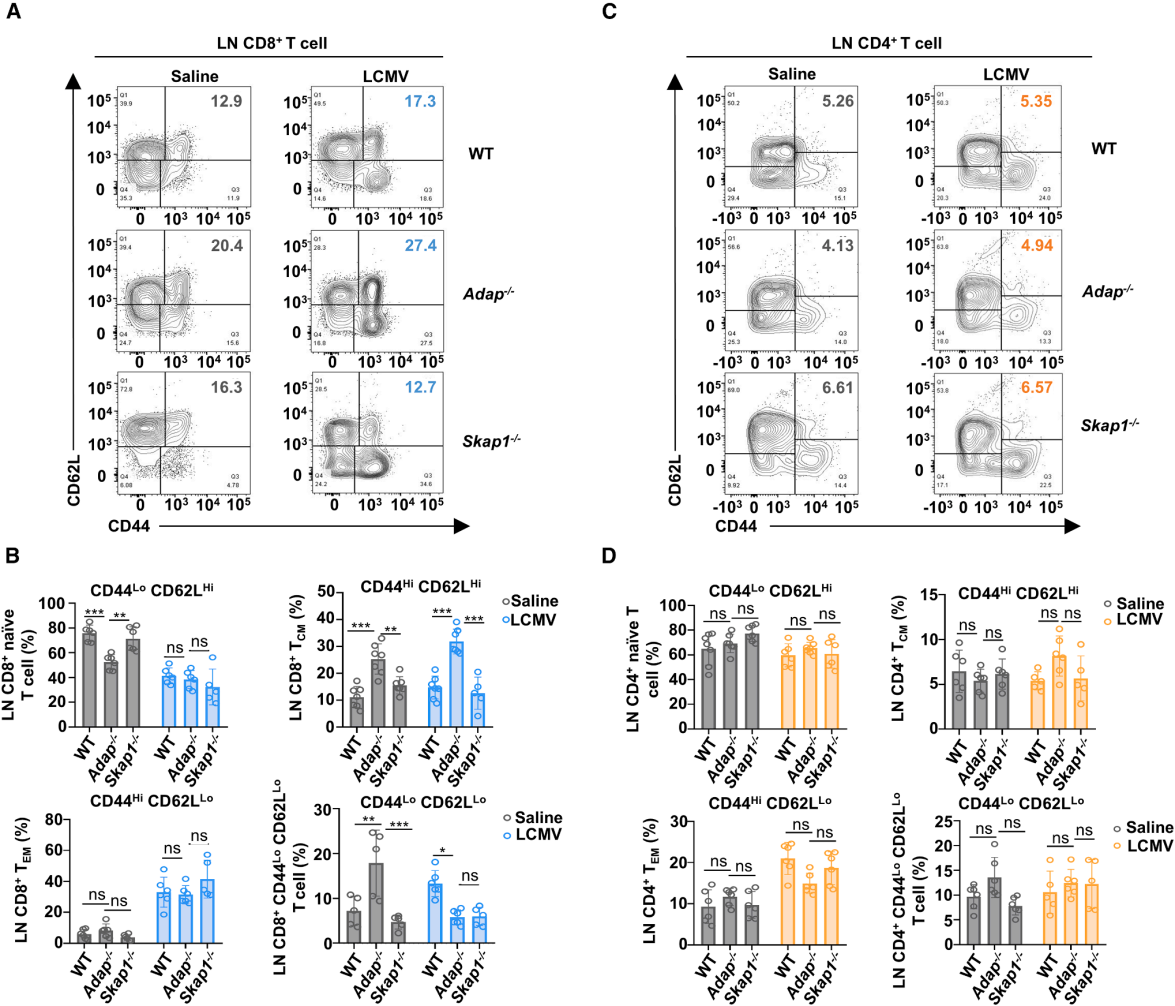
Increased CD8^+^ T_CM_-like T cells in the lymph nodes of ADAP-deficient mice under acute LCMV-Arm infection (A and B) Analysis of the lymph node CD8^+^ T cell subset differentiation on day 7 p.i. or in the saline control group. Representative contour plots showing the subsets of naive (CD44^Lo^ CD62L^Hi^), T_CM_ (CD44^Hi^ CD62L^Hi^), T_EM_ (CD44^Hi^ CD62L^Lo^), and CD44^Lo^ CD62L^Lo^ cells from WT, *Adap*^−/−^, and *Skap1*^−/−^ mice. The frequencies of the indicated subsets are shown in (B) (*n* ≥ 5 mice/group, two-way ANOVA and Bonferroni’s multiple comparison). (C and D) Analysis of the differentiation of the lymph node CD4^+^ T cell subsets on day 7 p.i. or the saline control. Representative contour plots showing the subsets of naive (CD44^Lo^ CD62L^Hi^), T_CM_ (CD44^Hi^ CD62L^Hi^), T_EM_ (CD44^Hi^ CD62L^Lo^), and CD44^Lo^ CD62L^Lo^ cells from WT, *Adap*^−/−^, and *Skap1*^−/−^ mice. The frequencies of the indicated subsets are shown in (D) (*n* ≥ 5 mice/group, two-way ANOVA, and Bonferroni’s multiple comparison). All data are expressed as mean with SD. *n* represents biological replicates per group. *∗p* < 0.05, *∗∗p* < 0.01, *∗∗∗p* < 0.001; ns, not significant.

To investigate whether ADAP-mediated differentiation of CD8^+^ T_CM_-like cells occurs during acute viral infection, we infected C57BL/6J WT, *Adap*^−/−^, and *Skap1*^−/−^ mice with the Armstrong strain of lymphocytic choriomeningitis virus (LCMV-Arm; 2 × 10^5^ plaque-forming units [PFU], i.p.), lymph nodes and spleens were collected on day 7 post-infection (p.i.) (Figure 2A). Successful infection was confirmed by marked splenomegaly (Figure 2B) and significant expansion of Gp33-specific CD8^+^ T cells in the spleens of WT, *Adap*^−/−^, and *Skap1*^−/−^ mice (Figures 2C and 2D). Notably, the frequency of antigen-specific effector CD8^+^ T cells was significantly increased in *Adap*^−/−^ mice compared with WT controls (Figures 2E and 2F). It was also higher than that in *Skap1*^−/−^ mice. These data suggest that the lack of ADAP enhances the clonal expansion of CD8^+^ T cells. Next, we assessed the percentages of CD8^+^ T_CM_-like cells in WT, *Adap*^−/−^ and *Skap1*^−/−^ mice on day 7 p.i. Compared with WT mice, *Adap*^−/−^ mice exhibited an even higher frequency of CD8^+^ but not CD4^+^ T_CM_-like cells in lymph nodes (Figures 3A–3D and S1A–S1D). However, the proportion of CD8^+^ T_CM_-like cells in *Skap1*^−/−^ mice was comparable to that in WT controls, suggesting a specific requirement for ADAP in the phenotypic development of the CD8^+^ T_CM_-like cell subset. Consistent with this observation, *Adap*^−/−^ mice also displayed an increased proportion of Gp33-specific CD8^+^ T_CM_-like T cells following acute LCMV-Arm infection (Figure S1E). Collectively, these findings demonstrate that ADAP deficiency promotes CD8^+^ T cell effector expansion and the acquisition of a T_CM_-like phenotype.

### ADAP-deficient CD8^+^ T cells exhibit enhanced effector function

**Figure 4:**
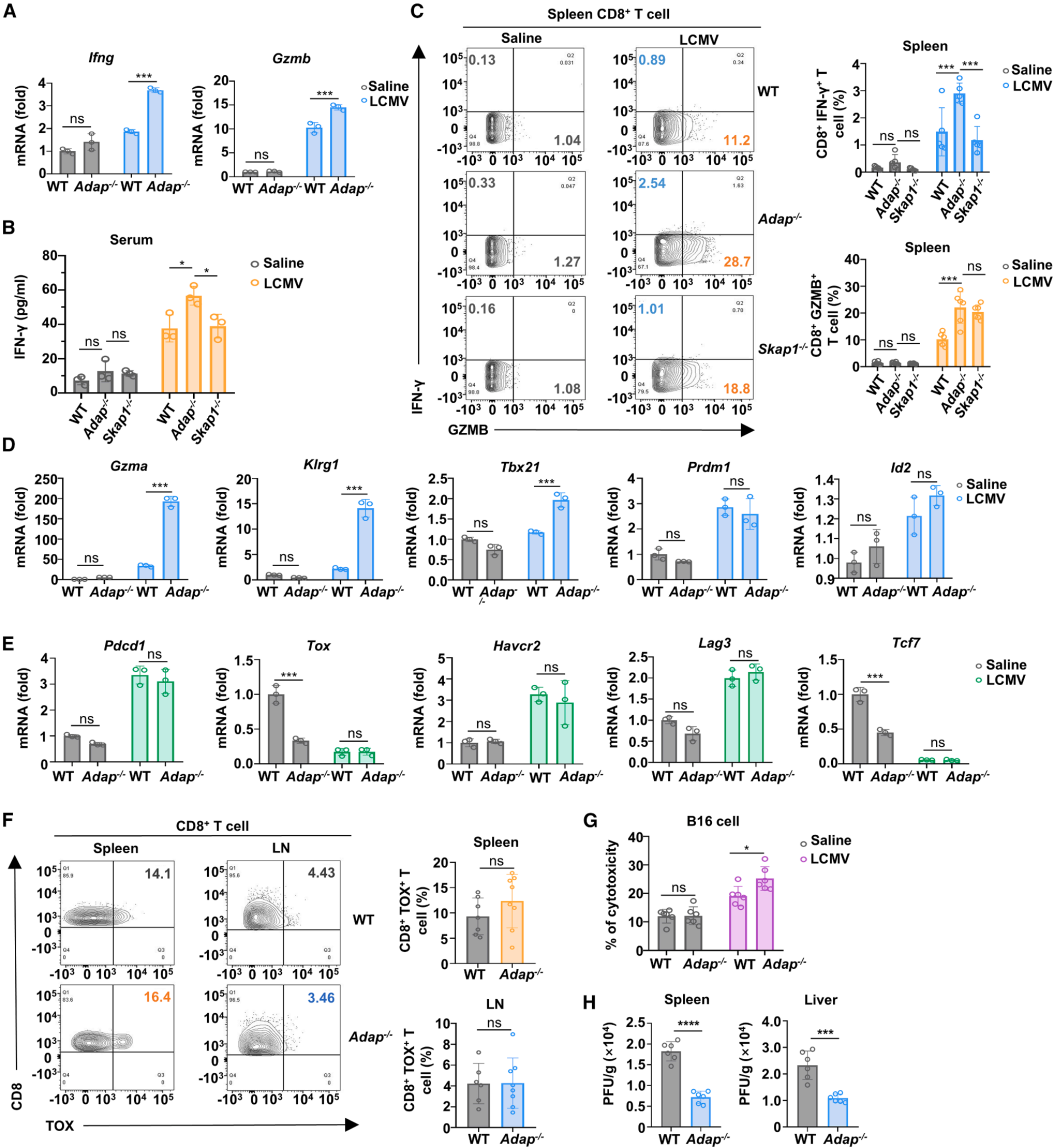
*Adap*^−/−^ CD8^+^ T cells display enhanced cytotoxic effects (A) RT-qPCR was used to detect the expression of *Ifn-γ* and *Gzmb* mRNA in purified splenic CD8^+^ T cells isolated from WT and *Adap*^−/−^ mice with or without LCMV-Arm infection (*n* = 3 mice/group, two-way ANOVA and Bonferroni’s multiple comparison). (B) The cytokine secretion levels of IFN-γ were measured by ELISA in the serum of WT, *Adap*^−/−^, and *Skap1*^−/−^ mice that were either mock-infected or infected with LCMV-Arm (*n* = 3 mice/group, two-way ANOVA and Bonferroni’s multiple comparison). (C) Production of cytokines, including IFN-γ^+^ and GZMB^+^ cells, represented as percentage of CD8^+^ T cells. Representative contour plots showing the production of IFN-γ and GZMB from splenic CD8^+^ T cells of WT, *Adap*^−/−^, and *Skap1*^−/−^ mice with or without infection. The frequency of the indicated subsets is shown on right (*n* = 6 mice/group, two-way ANOVA and Bonferroni’s multiple comparison). (D) Analysis of key regulatory genes expression of effector CD8^+^ T cells. Relative transcripts expression of effector CD8^+^ T cells from WT and *Adap*^−/−^ mice was measured by RT-qPCR on day 7 p.i. or in the saline control group (*n* = 3 mice/group, two-way ANOVA, and Bonferroni’s multiple comparison). (E) Analysis of key regulatory gene expression of exhausted CD8^+^ T cells. Relative transcript expression of exhausted CD8^+^ T cells from WT and *Adap*^−/−^ mice was measured by RT-qPCR on day 7 p.i. or in the saline control group (*n* = 3 mice/group, two-way ANOVA and Bonferroni’s multiple comparison). (F) Analysis of TOX expression in CD8^+^ T cells. Representative contour plots showing the expression of TOX in the spleens and the lymph nodes of WT and *Adap*^−/−^ mice uninfected or infected with LCMV-Arm (*n* ≥ 7 mice/group, unpaired Student’s *t* test). (G) Analysis of the cytotoxicity of splenic CD8^+^ T cells from WT and *Adap*^−/−^ mice infected with or without LCMV-Arm. Cytotoxicity was detected by using LDH assay (*n* = 6 mice/group, two-way ANOVA and Bonferroni’s multiple comparison). (H) Viral loads of LCMV-Arm in the spleens and the livers of WT and *Adap*^−/−^ mice shown on day 5 p.i. Tissue viral loads were determined by RT-qPCR (*n* = 6 mice/group, unpaired Student’s *t* test). All data are expressed as mean with SD. *n* represents biological replicates per group. *∗p* < 0.05, *∗∗∗p* < 0.001, ∗∗∗∗*p* < 0.0001; ns, not significant.

To evaluate whether ADAP regulates the effector function of CD8^+^ T cells in response to LCMV-Arm infection, we quantified the expression of *Ifng* and *Gzmb* in splenic CD8^+^ T cells. As shown in Figure 4A, while LCMV-Arm infection induced substantial upregulation of *Ifng* and *Gzmb* in both WT and *Adap*^−/−^ CD8^+^ T cells, *Adap*^−/−^ CD8^+^ T cells produced higher levels of these effector molecules than WT controls. Correspondingly, although serum ELISA revealed elevated IFN-γ secretion in WT, *Adap*^−/−^, and *Skap1*^−/−^ mice after infection, the increase was most pronounced in *Adap*^−/−^ mice (Figure 4B). Moreover, in line with this, *Adap*^−/−^ mice displayed a greater percentage of IFN-γ- and GZMB-expressing CD8^+^ T cells than WT and *Skap1*^−/−^ controls (Figure 4C). Considering the possibility of bystander activation in this context, cytokine production was further assessed in antigen-specific CD8^+^ T cells, which also exhibited increased proportions of IFN-γ- and GZMB-producing cells in the absence of ADAP (Figure S2). Consistent with the enhanced effector function, the expression of the cytotoxicity gene *Gzmb* and regulators of effector differentiation, such as *Klrg1* and *Tbx21*, was significantly increased in *Adap*^−/−^ CD8^+^ T cells compared with control cells (Figure 4D). Moreover, to determine whether ADAP deficiency influences the exhaustion state of CD8^+^ T cells during viral challenge, we analyzed exhaustion-associated gene expression. As shown in Figure 4E, the expression of key exhaustion-related genes (*Tox*, *Pdcd1*, *Lag3*, *Havcr2*, and *Tcf7*) was unchanged or slightly reduced in *Adap*^−/−^ CD8^+^ T cells relative to WT controls on day 7 p.i. Consistently, no significant difference was observed in the frequency of CD8^+^ TOX^+^ T cells (Figure 4F), and exhaustion marker expression remained comparable at day 30 p.i. (Figure S3), indicating that ADAP deficiency does not alter the exhaustion program. Functionally, splenic cells from *Adap*^−/−^ mice infected with LCMV-Arm exhibited enhanced cytotoxicity against B16 cells relative to those from WT controls (Figure 4G). To further strengthen our observation, we measured the viral titers after LCMV-Arm infection. Compared with WT mice, *Adap*^−/−^ mice presented improved viral clearance in both the spleen and the liver (Figure 4H). Taken together, these data demonstrate that the absence of ADAP enhances the antiviral effector function of CD8^+^ T cells.

### ADAP deficiency promotes CD8^+^ T cell effector function via upregulation of ZEB2

**Figure 5:**
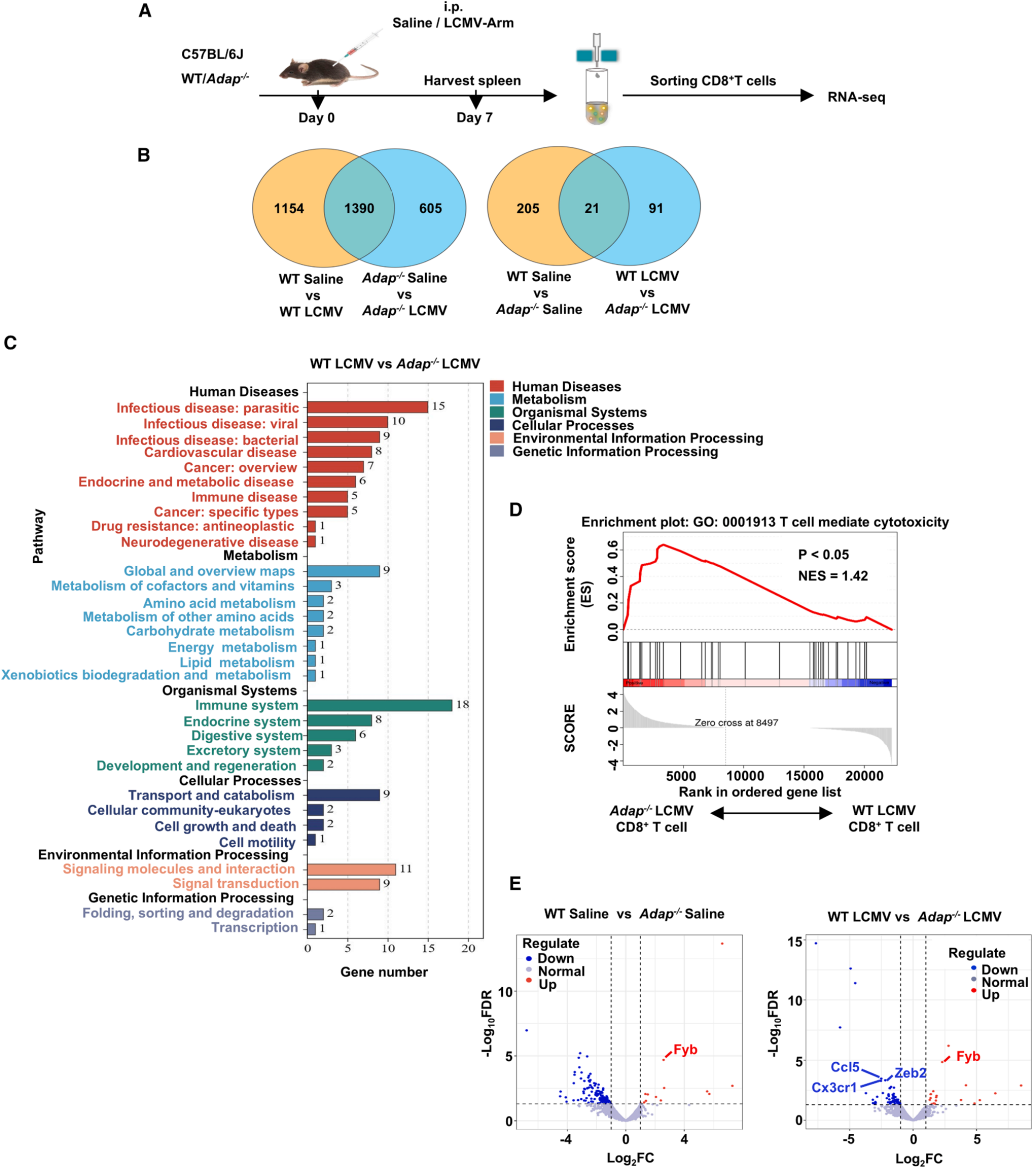
RNA-seq analysis revealed that the loss of ADAP drives the transcriptome switch of CD8^+^ T cells during LCMV-Arm infection with ZEB2 upregulation (A) Schematic diagram of the experimental design for RNA-seq of WT or *Adap*^−/−^ splenic CD8^+^ T cells. (B) Venn diagram of DEGs with an absolute log_2_ fold change ≥ 1/≤ −1 (false discovery rate [FDR] < 0.05) in mock-infected vs. LCMV-infected CD8^+^ T cells from WT mice compared with that from *Adap*^−/−^ mice (left), and DEGs with an absolute log_2_ fold change ≥ 1/≤ −1 (FDR < 0.05) in WT vs. *Adap*^−/−^ CD8^+^ T cells between mock-infected and LCMV-infected conditions (right). (C) KEGG biological pathway classification of the overlapping DEGs between CD8^+^ T cells from WT or *Adap*^−/−^ mice infected with LCMV-Arm. (D) GSEA enrichment plots showing normalized enrichment scores (NESs) for the pathways associated with T cell-mediated cytotoxicity using RNA-seq datasets from WT and *Adap*^−/−^ CD8^+^ T cells infected with LCMV-Arm. (E) Volcano plot of gene expression data with an absolute log_2_ fold change ≥ 1/≤ −1 (FDR < 0.05) comparing the WT vs. *Adap*^−/−^ splenic CD8^+^ T cells purified from mock-infected (left) or LCMV-Arm-infected (right) mice.

Given that ADAP plays a role in regulating the subset differentiation of CD8^+^ but not CD4^+^ T cell subsets, we sought to determine whether ADAP deficiency impacts the transcriptome switch of CD8^+^ T cells during LCMV-Arm infection. CD8^+^ T cells were sorted from the spleens of mock-infected or LCMV-Arm-infected WT and *Adap*^−/−^ mice on day 7 p.i. and subjected to RNA-seq (Figure 5A). To screen for potential target genes associated with ADAP deficiency-induced enhancement of effector function, we performed a comprehensive Venn diagram analysis on RNA-seq datasets, comparing gene expression profiles between WT saline and WT LCMV, as well as between *Adap*^−/−^ saline and *Adap*^−/−^ LCMV. Venn diagram decomposition revealed 1,390 shared differentially expressed genes (DEGs). Furthermore, we identified 1,154 genes that were specifically differentially expressed in the WT saline versus WT LCMV comparison and 605 genes that were uniquely differentially expressed in the *Adap*^−/−^ saline versus *Adap*^−/−^ LCMV comparison. Additionally, we conducted a separate Venn diagram analysis to compare gene expression between WT saline and *Adap*^−/−^ saline, as well as between WT LCMV and *Adap*^−/−^ LCMV. This analysis revealed that only 21 genes were commonly differentially expressed across these two sets of comparisons, with 205 genes uniquely expressed in the former set and 91 genes specifically expressed in the latter set (Figure 5B). When we performed the Kyoto Encyclopedia of Genes and Genomes (KEGG) enrichment analysis on the WT LCMV and *Adap*^−/−^ LCMV groups, significant differences in pathways were identified primarily within five categories: “human diseases,” “metabolism,” “organismal systems,” “cellular processes,” and “environmental information processing” (Figure 5C). Moreover, gene set enrichment analysis (GSEA) revealed that T cell cytotoxicity-related functions were more enriched in *Adap*^−/−^ CD8^+^ T cells than in WT CD8^+^ T cells (Figure 5D).

**Figure 6:**
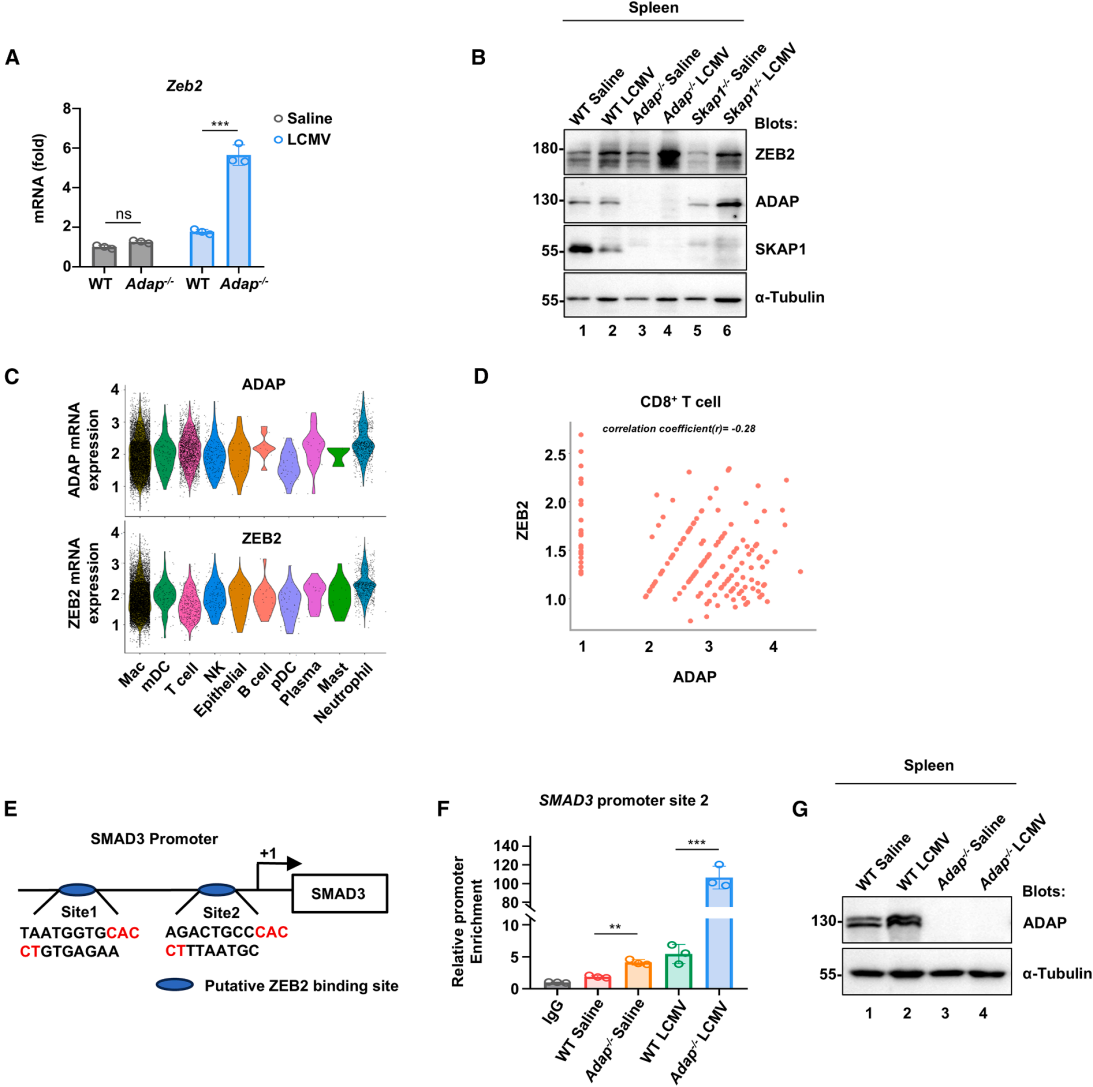
ADAP deficiency enhances the level of the transcription factor ZEB2 binding to the *Smad3* promoter (A) The expression of *Zeb2* in the splenic CD8^+^ T cells of WT and *Adap*^−/−^ mice, either uninfected or infected with LCMV-Arm, was measured by RT-qPCR assay (*n* = 3 mice/group, two-way ANOVA and Bonferroni’s multiple comparison). (B) Western blotting was used to analyze the expression of ZEB2 in WT, *Adap*^−/−^, and *Skap1*^−/−^ splenic cells either mock-infected or infected with LCMV-Arm. (*n* = 3 independent repeat experiments). (C) Expression profiles of ADAP and ZEB2 in different immune cells were analyzed by using the publicly available single-cell RNA-seq dataset GEO: GSE150861. (D) Scatterplot showing the correlations between ADAP and ZEB2 in CD8^+^ T cells from the GEO database (GEO: GSE150861). (E) The ZEB2 binding consensus motif (5′-CACCT-3′) is depicted, along with a schematic representation of the two putative ZEB2 binding sites within the promoter region of the mouse *Smad3* gene. (F) CUT&RUN-qPCR analysis was used to examine the enrichment of the *Smad3* promoter (site 2) by anti-ZEB2 antibody in the WT or *Adap*^−/−^ splenic cells from mock-infected or LCMV-Arm-infected mice (*n* = 3 mice/group, two-way ANOVA and Tukey’s multiple comparison). (G) ADAP expression in WT and *Adap*^−/−^ splenic cells either mock-infected or infected with LCMV-Arm. (*n* = 3 independent repeat experiments). All data are expressed as mean with SD. *n* represents biological replicates per group. ∗∗*p* < 0.01, ∗∗∗*p* < 0.001; ns, not significant.

To further identify downstream target genes that account for CD8^+^ T cell effector function, differential gene expression analysis was performed between WT and *Adap*^−/−^ CD8^+^ T cell subsets upon LCMV-Arm infection relative to mock infection. As shown in Figure 5E, while CD8^+^ T cells expressed a substantial set of genes at similar levels irrespective of ADAP, a total of 112 DEGs were identified between *Adap*^−/−^ and WT CD8^+^ T cells after LCMV-Arm infection. Of note, ZEB2, encoding a key transcriptional repressor involved in effector/memory T cell differentiation,^54^ was among the top 20 upregulated DEGs in ADAP-deficient CD8^+^ T cells. Consistent with the RNA-seq data, while ZEB2 expression was negligible in CD8^+^ T cells from mock-infected mice, it was substantially increased following LCMV-Arm infection. This upregulation was most pronounced in *Adap*^−/−^ mice at both the mRNA and protein levels (Figures 6A and 6B). This ADAP-dependent regulation appeared to be specific, as *Skap1*^−/−^ cells (with intact ADAP expression) showed no comparable enhancement of infection-induced ZEB2 expression.

To investigate the transcriptional coregulation of ADAP and ZEB2 in human immune responses, we reanalyzed COVID-19 patient-derived single-cell RNA-seq datasets from the GEO repository, which revealed the cell type-specific expression dynamics of both ADAP and ZEB2 across heterogeneous immune cell populations, including T cells, B cells, and natural killer (NK) cells (Figure 6C). A subsequent correlation analysis focused on the expression levels of ADAP and ZEB2 in CD8^+^ T cells sourced from this comprehensive dataset, which demonstrated a significant negative correlation between the two proteins (Figure 6D). Given that ZEB2/SIP1 interacts with R-Smad complexes to modulate the TGF-β signaling pathway,^36^ to assess whether ZEB2 can directly regulate the *Smad3* gene, prediction of transcription factor-binding sites in the promoter regions of *Smad3* using the JASPAR database identified two potential consensus motifs (5′ CACCT 3′) for ZEB2 binding within the *Smad3* promoter (Figure 6E). To verify that these sites are functional in the *Smad3* promoter, we performed CUT&RUN-qPCR assays on WT and *Adap*^−/−^ CD8^+^ T cells, showing that LCMV-Arm could effectively stimulate ZEB2 binding predominantly to site 2 of the *Smad3* promoter (Figure 6F). Notably, compared with WT CD8^+^ T cells, *Adap*^−/−^ CD8^+^ T cells displayed an enhanced ZEB2 binding to the *Smad3* promoter under LCMV-Arm infection conditions (Figures 6F and 6G). Together, these data indicate that ADAP deficiency leads to higher ZEB2 protein levels and increased occupancy at a canonical target promoter.

### ADAP enhances K48-linked ubiquitination of ZEB2 by recruiting TRIM21

**Figure 7:**
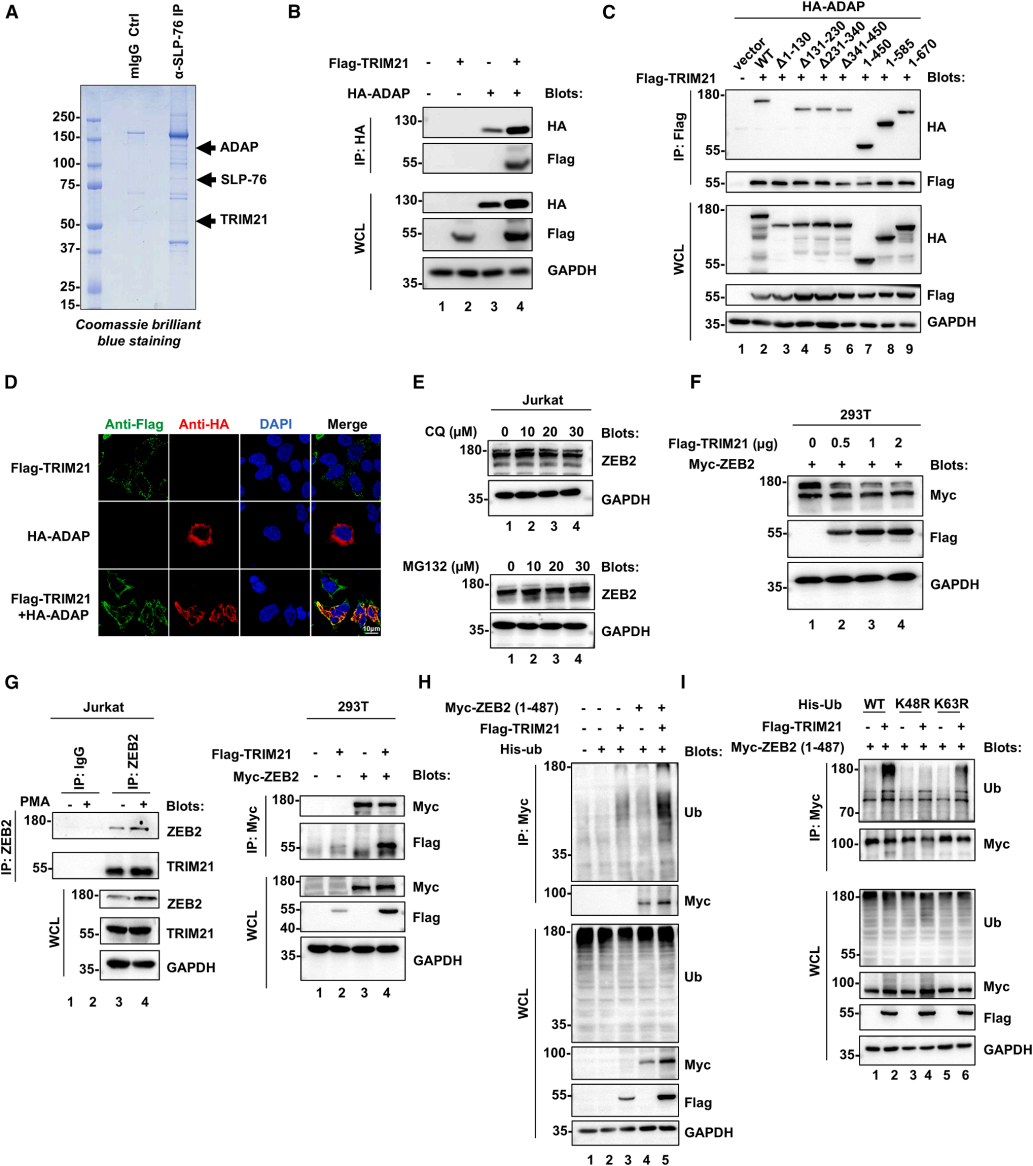
TRIM21 promotes ZEB2 degradation by increasing its K48-linked ubiquitination (A) Coomassie brilliant blue staining of SDS-PAGE gels showing SLP-76 immunoprecipitates from Jurkat cells. The proteins of interest are indicated by arrows. Representative Coomassie brilliant blue staining images of samples used for LC-MS analysis. (B) CoIP with an anti-HA antibody revealed the exogenous interaction of TRIM21 and ADAP in HEK293T cells (*n* = 3 independent repeat experiments). (C) HEK293T cells were co-transfected with Flag-tagged TRIM21 and HA-tagged full-length ADAP (FL) or the indicated mutants. Cell lysates were subjected to immunoprecipitation using anti-Flag beads, followed by immunoblotting with the indicated antibodies (*n* = 3 independent repeat experiments). Δ1-130, Δ131-230, Δ231-340, and Δ341-450 represent ADAP deletion mutants lacking amino acids 1–130, 131–230, 231–340, and 341–450, respectively. 1–450, 1–585, and 1–670 represent truncation mutants containing amino acids 1–450, 1–585, and 1–670, respectively. (D) Confocal microscopy was performed on TRIM21 (green) and ADAP (red) in HEK293T cells transfected with Flag-tagged TRIM21 and HA-tagged ADAP plasmids and subjected to an immunofluorescence assay (*n* = 3 independent repeat experiments). Scale bars,10 μm. (E) The protein expression levels of ZEB2 in Jurkat cells were assayed following treatment with increasing doses of CQ or MG132 for 6 h (*n* = 3 independent repeat experiments). (F) ZEB2 protein levels in HEK293T cells transfected with gradient concentrations of Flag-tagged TRIM21 (*n* = 3 independent repeat experiments). (G) Left: coIP with an anti-ZEB2 antibody in Jurkat cells revealed the endogenous interaction of TRIM21 and ZEB2. Right: coIP with an anti-Myc antibody revealed the interaction of exogenous TRIM21 and ZEB2 in HEK293T cells (*n* = 2 independent repeat experiments). (H) Ubiquitination of ZEB2 after TRIM21 overexpression in HEK293T cells (*n* = 3 independent repeat experiments). (I) The ubiquitination of ZEB2 in response to TRIM21 overexpression was investigated in HEK293T cells transfected with mutated His-Ub plasmids (K48R, only the Lys48 residue was mutated; K63R, only the Lys63 residue was mutated) (*n* = 3 independent repeat experiments). *n* represents biological replicates per group.

To elucidate the molecular mechanism underlying the ADAP-mediated regulation of ZEB2 expression, we performed liquid chromatography-tandem mass spectrometry (LC-MS/MS) in Jurkat T cells to identify ADAP-interacting proteins. This analysis revealed that TRIM21, a member of the tripartite motif (TRIM) family with known E3 ubiquitin ligase activity and roles in antiviral innate immunity,^55^ as a potential ADAP-associated partner (Figure 7A and Table S2). Co-immunoprecipitation (coIP) assays confirmed the exogenous interaction between TRIM21 and ADAP (Figure 7B). Domain mapping further localized the TRIM21-binding region to residues 1–130 of ADAP (Figure 7C). Immunofluorescence (IF) microscopy demonstrated cytoplasmic colocalization of TRIM21 and ADAP in HEK293T cells (Figure 7D), collectively establishing TRIM21 as a direct downstream effector of ADAP.

Given the elevated ZEB2 levels observed in ADAP-deficient cells during viral infection, we hypothesized that ADAP regulates ZEB2 stability via proteasomal degradation. To this end, we investigated the effect of ADAP on ZEB2 expression. Western blotting showed that ADAP overexpression dramatically accelerated ZEB2 protein degradation after treatment with the protein synthesis inhibitor cycloheximide (CHX), whereas the ADAP mutant Δ1–130 failed to affect ZEB2 stability, suggesting that ADAP could shorten the half-life of ZEB2 (Figures S4A and S4B). Treatment of Jurkat cells with a gradient concentration of the proteasome inhibitor MG132 but not the lysosomal inhibitor chloroquine (CQ), resulted in dose-dependent accumulation of ZEB2 protein (Figure 7E), confirming the proteasome-mediated regulation of the ZEB2 stability. Consistently, the ubiquitination of ZEB2 was enhanced by the overexpression of ADAP (Figure S4C). Together, these results suggest that ADAP regulates ZEB2 stability in a ubiquitin-dependent manner.

To investigate the role of TRIM21 in this process, we overexpressed TRIM21 and observed a corresponding reduction in exogenous ZEB2 protein levels (Figure 7F). CoIP analysis revealed both exogenous and endogenous interactions between TRIM21 and ZEB2 (Figure 7G). To map the binding sites in TRIM21 and ZEB2, we analyzed the interaction between Myc-tagged ZEB2 and Flag-tagged full-length TRIM21 and its truncation mutants. Mapping assays revealed that the PRY-SPRY domain was the main fragment of TRIM21 responsible for binding to ZEB2 (Figures S5A and S5B). Critically, TRIM21 overexpression significantly promoted ZEB2 ubiquitination (Figure 7H), with specificity for K48-linked polyubiquitin chains (Figure 7I). To determine whether the ubiquitinase domain of TRIM21 is required for ZEB2 ubiquitination, we generated a previously reported TRIM21 RING domain deletion (ΔRING) mutant.^39^ We found that overexpression of the ΔRING mutant failed to induce ZEB2 degradation (Figure S5C), demonstrating that the RING domain is required for the E3 ligase activity of TRIM21.

As the SMAD-binding domain (SBD) of ZEB2 has been shown to be critical for its ubiquitination,^56^ we next constructed an SBD deletion (ΔSBD) mutant of ZEB2. Notably, the ΔSBD mutation markedly reduced ZEB2 ubiquitination (Figure S5D), suggesting that the SBD is indispensable for TRIM21-mediated ubiquitination of ZEB2. To identify the potential ubiquitination sites on ZEB2, we analyzed the distribution of lysine (K) residues within the SBD region of ZEB2 using the UniProt database and generated a series of site-directed mutants (K462O, K475O, K479O, K485O, and K487O). When these mutants were co-expressed with TRIM21 and His-Ub in HEK293T cells, TRIM21 substantially enhanced the ubiquitination of Myc-ZEB2 (WT), whereas modest increases were observed for the K475O mutant (Figure S5E). Taken together, these results indicate that TRIM21 mediates the K48-linked ubiquitination of ZEB2 at lysine residue K475.

## Discussion

The acute effector response of CD8^+^ T cells is crucial for pathogen clearance and profoundly shapes the quantity and quality of memory CD8^+^ T cells, which are key determinants of robust immunity upon pathogen re-exposure.^57^ Here, we observed that the loss of ADAP promoted effector expansion and the differentiation of T_CM_-like CD8^+^ T cells (CD44^Hi^ CD62L^Hi^) via recruiting TRIM21 to enhance K48-linked polyubiquitination and degradation of the transcription factor ZEB2.

ADAP serves as a negative regulator of effector and memory-like CD8^+^ T cells, as *Adap*^−/−^ mice harbor elevated CD44^Hi^ CD122^Hi^ CD8^+^ T cells under steady-state and pathogenic conditions.^51^^,^^52^ Extending these observations, we found that LCMV-Arm infection triggered a pronounced expansion of Gp33-specific CD8^+^ T cells (Figures 2C and 2D) and the generation of CD8^+^ CD44^Hi^ CD62L^Hi^ T_CM_ memory-like T cells of *Adap*^−/−^ mice compared with those in WT controls (Figures 3A and 3B). This T_CM_-like CD8^+^ T cell formation aligns with increased T_CM_-like cell frequencies in uninfected *Adap*^−/−^ mice (Figure 1C), suggesting a conserved regulatory role for ADAP across physiological and pathological contexts. Functionally, ADAP deficiency upregulates the production of cytotoxic mediators (GZMA, GZMB, and IFN-γ) and expression of transcription factors (TBX21 and KLRG1) (Figures 4A–4D). This ADAP-deficiency-driven augmentation of CD8^+^ T cell response led to a reduction in viral loads in both the spleen and the liver (Figure 4H), suggesting that ADAP has a proviral activity for acute LCMV infection. This is consistent with a previous report that ADAP facilitates the infection of HIV in a T cell-dependent manner,^43^ wherein ADAP facilitates virological synapse formation, thereby promoting cell-to-cell viral spread.^43^ However, it was reported that ADAP is also a critical host protective factor against H5N1.^58^ Importantly, we recently showed ADAP is an interferon-stimulated gene (ISG) and ADAP deficiency in macrophages impairs the type I interferon response to Sev and vesicular stomatitis virus (VSV).^59^ Furthermore, we demonstrated in macrophages that ADAP acts as an intracellular immune checkpoint, negatively regulating inflammatory responses.^53^^,^^59^ Thus, ADAP serves as a broad spectrum, cell-intrinsic brake across multiple immune lineages and plays a complex, context-dependent role in viral infections. As in the case of HIV infection, the current study favors the model that during acute LCMV-Arm infection, ADAP deficiency enhances viral clearance and the acquisition of a T_CM_-like phenotype of CD8^+^ T cells. One possible reason is that both LCMV (used in this study) and HIV are RNA viruses that have evolved sophisticated mechanisms to evade the host immune system, and CD8^+^ T cells play a dominant role in controlling the replication of these viruses. In this context, modulation of ADAP signaling may influence the quality of antiviral T cell immunity. While ADAP deficiency enhanced CD8^+^ T cell cytotoxicity, it did not exacerbate the expression of exhaustion markers (Figures 4E and 4F), implying that ADAP specifically influences effector-memory differentiation rather than exhaustion. These findings highlight that, rather than uniformly promoting antiviral responses, ADAP appears to differentially regulate innate and adaptive immunity, and the net outcome depends on the relative contribution of these compartments in a given infection setting. Future studies employing cell type-specific deletion of ADAP will be important to further dissect its precise roles in distinct immune populations.

**Figure 8:**
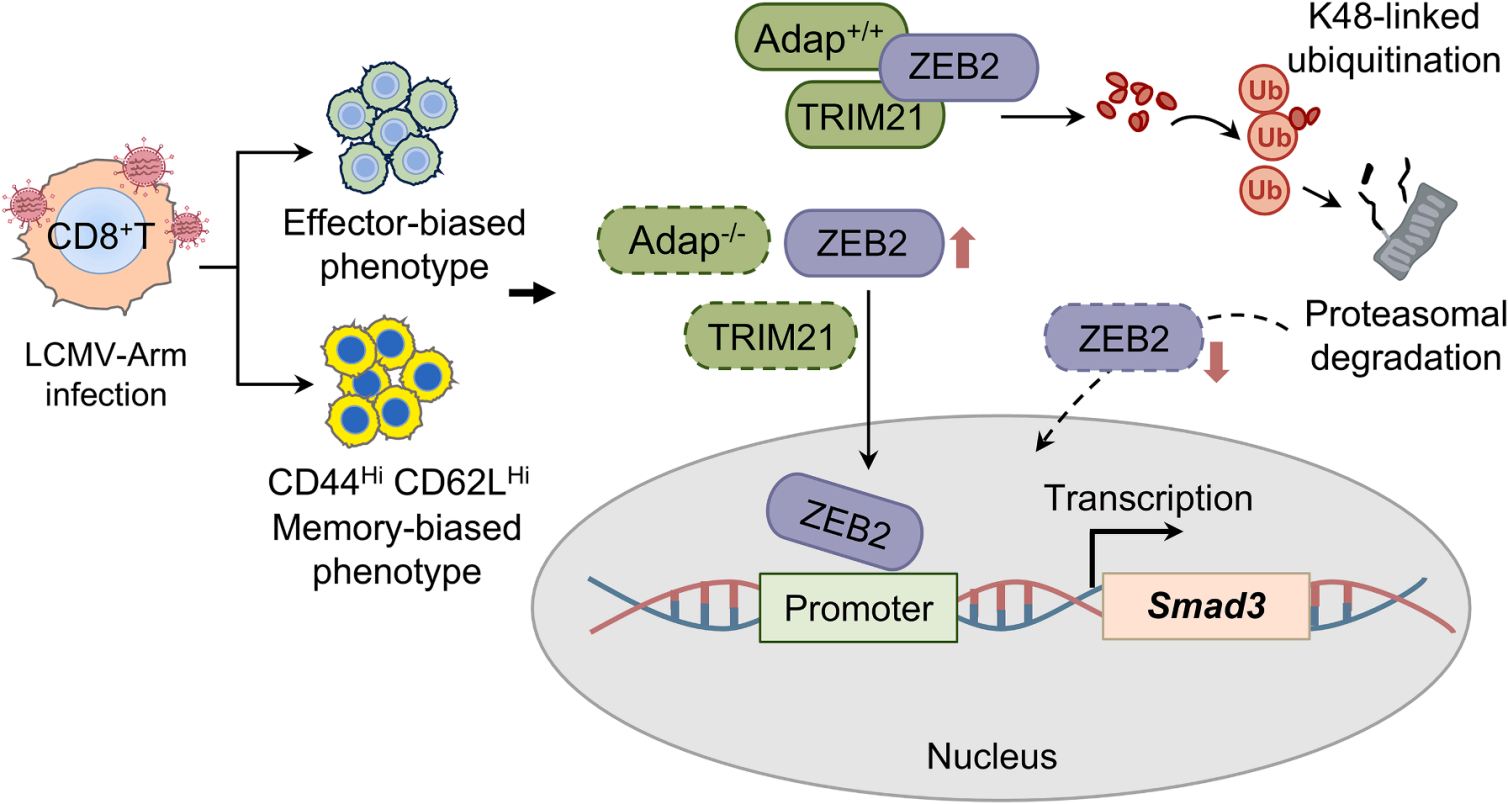
A model for how ADAP regulates CD8^+^ T cell fate decisions by controlling TRIM21-mediated ZEB2 ubiquitination and degradation In wild-type CD8^+^ T cells, ADAP recruits the E3 ubiquitin ligase TRIM21 to ZEB2, limiting ZEB2 levels via TRIM21-mediated K48-linked polyubiquitination (K48-Ub) and proteasomal degradation. During acute LCMV-Arm infection, when ADAP is under-expressed or deficient in CD8^+^ T cells, this restraint on ZEB2 is released, leading to increased ZEB2 protein. Elevated ZEB2 exhibits enhanced binding to the promoters of target genes, such as *Smad3* and potentially others, which correlates with enhanced effector expansion and increased central memory-like differentiation (CD44^Hi^ CD62L^Hi^) of CD8^+^ T cells.

ZEB2 is an E-box-binding transcription repressor that plays a pivotal role in balancing effector and memory differentiation of CD8^+^ T cells.^60^ Our RNA-seq analysis identified ZEB2 as one of the most significantly altered transcriptional regulators in *Adap*^−/−^ CD8^+^ T cells (Figure 5E), and loss of ADAP significantly enhanced ZEB2 expression (Figures 6A and 6B). Mechanistically, we found that ADAP interacts with the E3 ubiquitin ligase TRIM21 and regulates ZEB2 stability via TRIM21-mediated K48-linked ubiquitination of ZEB2 (Figure 7I), which appeared to be phosphorylation-independent (Figure 7H). This is consistent with the notion that ZEB2 stability is tightly regulated by ubiquitination. Similar regulatory mechanisms have been reported wherein DGKG recruits USP16 to deubiquitinate ZEB2 and sustain TGF-β1 signaling in tumor angiogenesis,^61^ and FBXW7 and FBXO45 promote ZEB2 degradation in a phosphorylation-dependent or SPRY domain-mediated manner.^56^^,^^62^ A recent study by Shi et al.^41^ established a pivotal role for TRIM21 in restraining CD8^+^ T cell antitumor activity by stabilizing PD-1 through K63-linked ubiquitination, thereby modulating immune checkpoint responsiveness. While that work positioned TRIM21 as a regulator of T cell exhaustion at the level of surface receptor stability, our findings extend this concept by uncovering a role for TRIM21 in controlling the intrinsic transcriptional program of CD8^+^ T cells. Specifically, our data support a model in which ADAP acts as a critical upstream adapter that recruits TRIM21 to promote ZEB2 ubiquitination, which in turn correlates with suppressed effector function and reduced central memory-like differentiation of CD8^+^ T cells (Figure 8). Our CUT&RUN-qPCR assays confirmed that ADAP deficiency increases ZEB2 occupancy at the *Smad3* promoter (Figures 6F and 6G), consistent with higher ZEB2 protein levels. However, the functional consequences of this enhanced binding on *Smad3* transcription or Smad3 acivity remains unknown. Furthermore, the enhanced effector expansion and central memory-like differentiation in *Adap*^−/−^ CD8^+^ T cells may or may not be mediated through Smad3, given ZEB2 has broad transcriptional network. Future studies are needed to identify the exact downstream effectors of this axis that are specifically responsible for regulating CD8^+^ T cell fate.

Collectively, our findings reveal ADAP as a critical orchestrator of CD8^+^ T cell responses, where its interaction with TRIM21 controls ZEB2 stability to balance effector and memory differentiation. This axis represents a promising therapeutic target for enhancing antiviral and antitumor immunity. Future studies exploring pharmacological or genetic manipulation of this pathway may yield innovative strategies to optimize T cell responses in infections and cancer.

### Limitations of the study

While this study identified ADAP-TRIM21-ZEB2 as a novel axis regulating CD8^+^ T cell differentiation, several limitations should be noted. First, the global *Adap*^−/−^ mouse model may introduce developmental or non-T cell confounders. Adoptive transfers or bone marrow chimeras are needed to confirm cell-intrinsic mechanisms. Additionally, the molecular mechanisms by which ADAP regulates the transcriptional activity of ZEB2 remain unclear. Finally, whether this increased binding affects *Smad3* transcription or Smad3 activity, and whether the phenotypic changes in CD8^+^ T cells are mediated specifically through Smad3 versus other ZEB2 targets, requires further investigation.

## Resource availability

### Lead contact

Further information and requests for resources and reagents should be directed to and will be fulfilled by the lead contact, Hebin Liu (Hebin.Liu@nottingham.edu.cn).

### Materials availability

This study did not generate new unique reagents.

### Data and code availability

• The raw RNA-seq data are available from the Gene Expression Omnibus (GEO: GSE300099). The uncropped western blot images are provided as supplemental information.
• This paper does not report the original code.
• Any additional information required to reanalyze the data reported in this paper is available from the lead contact upon request.

## Acknowledgments

This work was supported by grants from Natural Science Foundation of Jiangsu Higher Education Institution-Key Program under 21KJA310002, Soochow University Research Development Funds under Q424900220, National Natural Science Foundation of China (NSFC) under grant no. 31470840, and the Priority Academic Program Development of Jiangsu Higher Education Institutions.

## Author contributions

Conceptualization, H.L.; data curation, J.L., Y.L., F.Y., P.Z., and T.J.; formal analysis, J.L., F.Y., Y.L., P.Z., T.J., and H.L.; funding acquisition, H.L.; investigation, J.L., F.Y., Y.L., P.Z., and T.J.; methodology, J.L., F.Y., Y.L., P.Z., T.J., and H.L.; project administration, Y.L.; resources, P.Z. and T.J.; supervision, H.L.; validation, J.L., F.Y., Y.L., P.Z., T.J., and H.L.; writing – original draft, Y.L., J.L., and H.L.; writing – review and editing, Y.L., J.L., F.Y., and H.L.

## Declaration of interests

The authors declare no competing interests.

## STAR★Methods

### Key resources table

**Table:** 

REAGENT or RESOURCE	SOURCE	IDENTIFIER
**Antibodies**		

rabbit anti-ADAP	Millipore	Cat#: 07–546; RRID: AB_310704
rabbit anti-α-Tubulin	Abcam	Cat#: ab4074; RRID: AB_2288001
mouse anti-ZEB2	Proteintech	Cat#: 67514-1-Ig; RRID: AB_2882735
rabbit anti-SKAP55	Proteintech	Cat#: 19247-1-AP; RRID: AB_10859099
mouse anti-Flag	Proteintech	Cat#: 66008-4-Ig; RRID: AB_2918475
mouse anti-HA	Proteintech	Cat#: 66006-2-Ig; RRID: AB_2881490
rabbit anti-TRIM21	CST	Cat#: 92043; RRID: AB_2800177
rabbit anti-Myc	Proteintech	Cat#: 16286-1-AP; RRID: AB_11182162
mouse anti-ubiquitin (P4D1)	CST	Cat#: 3936; RRID: AB_331292
mouse anti-GAPDH	Proteintech	Cat#: 60004-1-Ig; RRID: AB_2107436
HRP-conjugated goat anti-rabbit IgG (H + L)	CST	Cat#: 7074; RRID: AB_2099233
HRP-conjugated goat anti-mouse IgG (H + L)	CST	Cat#: 91196; RRID: AB_2940774
Alexa Fluor 488-conjugated anti-mouse IgG (H + L), F (ab’)_2_ Fragment	CST	Cat#: 4408S; RRID: AB_10694704
Alexa Fluor 555-conjugated anti-rabbit IgG (H + L), F (ab’)_2_ Fragment	CST	Cat#: 4413S; RRID: AB_10694110
FITC-conjugated anti-mouse CD3	BD Bioscience	Cat#: 555274; RRID: AB_395698
PE-conjugated anti-mouse CD8a	BD Bioscience	Cat#: 553032; RRID: AB_394570
Percp-Cy5.5-conjugated anti-mouse CD44	BD Bioscience	Cat#: 560570; RRID: AB_1727486
APC-conjugated anti-mouse CD62L	BD Bioscience	Cat#: 553152; RRID: AB_398533
eF660-conjugated anti-mouse TOX	Thermo Fisher	Cat#: 50-6502-82; RRID: AB_2574265
APC-Cy7-conjugated anti-mouse IFN-γ	BD Bioscience	Cat#: 561479; RRID: AB_10898181
PE-cy7-conjugated anti-mouse GZMB	Thermo Fisher	Cat#: 25-8898-82; RRID: AB_10853339
APC GP33-41 Tetramer	Cancer Research Center of Xiamen University	N/A

**Bacteria and virus strains**		

LCMV-Armstrong	Prof. Jianfeng Dai (Soochow University)	N/A

**Chemicals, peptides, and recombinant proteins**		

CHX	MCE	HY-12320
MG132	Sigma-Aldrich	M8699
CQ	Sigma-Aldrich	C6628
DMEM	BasalMedia	L110KJ
RPMI1640	BasalMedia	L210KJ
Fetal Bovine Serum (FBS)	Biochannel	BC-SE-FBS07
Penicillin/Streptomycin	NCM	C100C5
4% PFA	Beyotime	P0099
PMA/TPA	Beyotime	S1819
PMSF	Fdbio	FD0100
Hieff Trans® Liposomal 2000 transfection reagent	Yeasen	40802ES02
Hieff® qPCR SYBR Green Master Mix	Yeasen	11202ES03

**Critical commercial assays**		

LDH Cytotoxicity Assay Kit	Beyotime	C0016
CUT & RUN Assay kit	Vazyme	HD101
CD8a^+^ T cell Isolation Kit	Miltenyi	130-104-075
Murine IFN-γ ELISA Kit	Biorbyt	orb2705235
IC Fixation Buffer kit	eBioscience	88-8824-00
Hifair® III 1st Strand cDNA Synthesis SuperMix for qPCR	Yeasen	11141ES10

**Deposited data**		

Raw and analyzed data	This paper	GEO: GSE300099

**Experimental models: Cell lines**		

HEK293T	ATCC	CRL-3216
Jurkat, Clone E6-1	ATCC	TIB-152
BHK-21	ATCC	CCL-10

**Experimental models: Organisms/strains**		

Mouse: WT C57BL/6J	GemPharmatech	RRID: IMSR_GPT: N000295
Mouse: *Adap*^−/−^	Peterson et al.^45^	N/A
Mouse: *Skap1*^−/−^	Peterson et al.^45^	N/A

**Oligonucleotides**		

Primers for RT-qPCR, see Table S1	This paper	N/A

**Recombinant DNA**		

Plasmid: pSRα-HA-ADAP	Xiong et al.^49^	N/A
Plasmid: pCMV-ZEB2-Myc	Miaoling	P50954
Plasmid: pEnCMV-TRIM21-3 × Flag	Miaoling	P33677
Plasmid: pCMV-6 × His-UB	Miaoling	P50527
Plasmid: pCDNA3.1-6 × His-Ubiquitin-K48R	Miaoling	P7778
Plasmid: pCDNA3.1-6 × His-Ubiquitin-K63R	Miaoling	P7779

**Software and algorithms**		

FlowJo	Tree Star	V10
Graphpad Prism	Graphpad	V8.0.1

### Experimental model and study participant details

#### Animals

*Adap*^−/−^ and *Skap1*^−/−^ mice were kind gifts from Dr. C.E. Rudd (University of Cambridge, Cambridge, UK), which were originally generated by Peterson et al.^45^ Wild-type (WT) C57BL/6J mice were obtained from GemPharmatech (Nanjing, China) and used as controls for KO animals. All the mice were bred under specific pathogen-free conditions at the Animal Center of Soochow University. All animal studies were approved by the Animal Ethics Committee of Soochow University. Sex-matched male mice aged between 6 and 8 weeks were used. All animal experiments involved in this study were approved by the Ethics Committee of Soochow University (ECSU) (Approval No. SUDA202406A0266) and carried out in accordance with the approved guidelines. Only male mice were used in this study. As no cross sex comparative analyses were performed, the potential influence of sex on the findings could not be evaluated, which represents a limitation regarding the generalizability of the conclusions.

#### Cell lines and virus

HEK293T and Jurkat cell lines were purchased from the American Type Culture Collection (ATCC, Manassas, VA). BHK-21 cells were kindly provided by Prof. Jianfeng Dai (Soochow University). HEK293T and BHK-21 cells were maintained in DMEM supplemented with 10% FBS (Serana Europe, Pessin, Germany) and 100 U/mL penicillin/streptomycin (Beyotime). Jurkat cell lines were cultured in complete RPMI 1640 medium. All cell lines were authenticated by STR profiling and tested negative for mycoplasma contamination.

The LCMV-Armstrong viral strains were kindly provided by Prof. Jianfeng Dai (Soochow University) and were grown in BHK-21 cells as previously described.^63^ The infected BHK21 cells were incubated at 37°C in a humidified atmosphere with 5% CO_2_. The supernatants were purified. The viral RNA levels in the supernatants were quantified via RT-qPCR. The supernatants were then aliquoted and stored at −80°C until use. All the sequences of culture strains were sequenced with Oxford Nanopore technology (Agilent Technologies®) using the Artic protocol.

### Method details

#### Plasmids and transfection

The mammalian expression plasmid pSRα-HA-ADAP has been described previously.^49^ pCMV-ZEB2-Myc, pEnCMV-TRIM21-3 × Flag, pCMV-His-UB, pCMV-His-Ub-K48R and pCMV-His-Ub-K63R were purchased from Miaoling Bio. Transfection of HEK293T cells was performed via Hieff Trans® Liposomal 2000 Transfection Reagent according to the manufacturer’s instructions (Yeasen Biotechnology).

#### In *vitro* killing assay

B16 melanoma cells were pretreated with LCMV-Armstrong virus for 2 h. For the experimental groups, spleen cells and virus-treated B16 cells were cocultured at a ratio of 90:1 (effector: target) in 96-well plates. Parallel control groups containing either spleen cells alone or B16 cells alone were maintained at equivalent cell densities. All cultures were incubated at 37°C for 24 h in a humidified 5% CO_2_ atmosphere. After incubation, 20 μL of LDH release reagent was added to the high release control wells (maximum lysis control). After an additional incubation at 37°C for 1 h, the plates were centrifuged at 500 × g for 5 min at room temperature. Subsequently, 120 μL of the supernatant from each well was carefully transferred to a new 96 well plate. Lactate dehydrogenase (LDH) activity was quantified by adding 60 μL of LDH detection solution to each well, followed by a 30-min incubation on a dark rotary shaker at room temperature. After reading the optical density at 490 nm using a microplate reader, the degree of cytotoxicity was calculated according to the following equation^64^:

Cytotoxicity(%)=(Experientalgroup−spleencellonly)/(highreleasegroup−spleencellonly)×100%

#### Viral infection

Six-to eight-week-old sex-matched WT, *Adap*^−/−^ and *Skap1*^−/−^ C57BL/6J mice were injected with 2 × 10^5^ plaque-forming units (PFU) of LCMV-Armstrong intraperitoneally (i.p.), as described previously.^65^ On day 7 post-infection, the lymph nodes and spleens were harvested and analyzed by flow cytometry. Viral titers of the stocks were determined by plaque assays on Vero cell monolayers.^63^

#### Flow cytometry

A single cell suspension was prepared and washed with FACS buffer (2% FCS, 2 mM EDTA in PBS). The cells were stained with indicated antibodies for 30 min and fixed in 4% paraformaldehyde. Data were acquired on a FACSCanto II flow cytometer and analyzed using FlowJo software (TreeStar).

#### Tetramer assay

A single cell suspension was prepared from the spleens and lymph nodes by sieving and pipetting, followed by lysis with ACK buffer for 5 min and washing with FACS buffer (2% FCS, 2 mM EDTA in PBS). The cells were then stained with the H2-Db:GP33-41 tetramer for 1 h at 4°C in the dark. After washing once with precooled FACS buffer, the cells were stained with flow cytometry antibodies, such as PE-conjugated anti-CD8 and FITC-conjugated anti-CD3 antibodies, at 4°C in the dark for 30 min. The cells were washed twice with pre-cooled FACS buffer and resuspended in 500 μL of precooled FACS buffer. Data were acquired on a FACScanto II flow cytometer and analyzed using FlowJo software (TreeStar).

#### Quantitative real-time PCR

Total RNA was isolated from splenocytes using TRIzol reagent (Sigma-Aldrich) according to the manufacturer’s instructions. The first-strand cDNA was synthesized using Hifair II First Strand cDNA Synthesis Kit (Yeasen Biotechnology), and quantitative real-time PCR was carried out using the Hieff Quantitative PCR SYBR Green Master Mix (Yeasen Biotechnology) on the QuantStudio Design and Analysis System (Applied Biosystems, Foster City, CA). Relative expression levels were calculated using the standard ΔΔC_T_ method and normalized to the housekeeping gene. Primers were synthesized by Sangon Biotech (Shanghai, China).

#### Bioinformatics analysis

Gene expression datasets were obtained from the GEO database (GEO: GSE150861) of the National Center for Biotechnology Information. Expression data were analyzed using the GEO2R online tool. We downloaded the GEO: GSE150861 dataset, which includes scRNA-seq data from two COVID-19 patients. For differential expression analysis, the expression patterns of ADAP (FYB1) and ZEB2 were quantified across immune cell subsets. Violin plots were generated using the ggplot2 R package (v3.4.2) to visualize gene expression distributions. Cell-type-specific coexpression analysis of ADAP and ZEB2 in CD8^+^ T cells was performed by calculating Spearman’s correlation coefficients. Statistical significance was determined using Wilcoxon rank-sum tests with Benjamini-Hochberg correction for multiple comparisons.

#### RNA-sequencing analysis

CD8^+^ T cells were isolated from the spleens of WT and *Adap*^−/−^ mice in both the physiological saline group and the LCMV-Arm infection group using the CD8^+^ T cell sorting kit. Total RNA was extracted using TRIzol reagent (Sigma, Cat# T9424). The integrity and quantity of RNA were examined with an Agilent Bioanalyzer 2100 (Agilent Technologies). A sequencing library was prepared using the NEBNext Ultra RNA Library Prep Kit for Illumina (New England Biolabs) according to the manufacturer’s instructions. RNA-seq was performed by Gene *Denovo* Biotechnology Co. (Guangzhou, China). Differentially expressed genes between WT and *Adap*^−/−^CD8^+^ T cells were quantified using DESeq2 (version 1.20.0). Bioinformatic analysis was performed using OmicsMart, a dynamic real-time interactive online platform for data analysis (http://www.omicsmart.com). Venn diagrams and volcano plot were generated. A fold change greater than two and an FDR value <0.05 were used for identifying differentially expressed genes (DEGs). KEGG pathway analysis was performed to determine the significant functions and pathways of the DEGs. Normalized read counts of all expressed genes were subjected to gene set enrichment analysis (GSEA). In GSEA analysis, pathways with *p* values <0.05 were considered significantly enriched.

#### Immunoblotting and immunoprecipitation

Cell lysis, immunoprecipitation, and detection were performed as described previously.^66^ Briefly, cells were lysed in lysis buffer (1% Triton X-100 (v/v) in 20 mM Tris-HCl (pH 8.3) and 150 mM NaCl) supplemented with an EDTA-free protease inhibitor cocktail (Roche). For immunoprecipitation assays, cell lysates were precleared with Protein G Sepharose beads (GE Healthcare, Cat# 17061801), followed by incubation with antibodies at 4°C overnight. Beads were washed with 0.5 × lysis buffer three times and boiled in sample buffer to dissociate proteins. Immunoprecipitates were resolved by SDS-PAGE, and immunoblotting was performed with the indicated primary antibodies. Immunoblots were developed with HRP-conjugated secondary antibodies and visualized with enhanced chemiluminescence (ECL) reagents (Yeasen, Cat# 36208ES76).

#### Liquid chromatography/mass spectrometry analysis

Cell lysates and precipitates were prepared as described in immunoblotting and immunoprecipitation. Proteins of interest were dissociated from beads with 8 M urea, followed by reduction, alkylation and digestion according to standard protocols. Tryptic extracts were collected, lyophilized and resuspended in 0.1% formic acid, and analyzed using a Linear Ion Trap Orbitrap mass spectrometer (LTQ Orbitrap Elite ETD) (ThermoFisher) for liquid chromatography-tandem mass spectrometry (LC-MS/MS) analysis.

#### Confocal microscopy

Confocal microscopy was used to observe the colocalization of ADAP and TRIM21. HEK293T cells were co-transfected with HA-ADAP and Flag-TRIM21. Twenty-four hours post-transfection, the samples were fixed in 4% paraformaldehyde for 10 min at room temperature. Cells were permeabilized with 0.4% Triton X-100 in PBS for 10 min and blocked with 5% goat serum for 1 h, and then incubated with the indicated primary and secondary antibodies. Nuclei were counterstained with 4′, 6-diamidino-2-phenylindole (DAPI) for 10 min and then the cells were mounted with antifade mounting medium (Beyotime) and observed with a Zeiss confocal microscope (LSM800, Zeiss). The fluorescence intensity profile of the indicated proteins was quantified and processed using ImageJ.

#### Cleavage under targets and release using nuclease assay

CUT & RUN assays were performed using the Hyperactive pG-MNase CUT & RUN Assay Kit for PCR/qPCR (Vazyme, Cat# HD101), according to the manufacturer’s instructions. Briefly, 1 × 10^6^ spleen cells isolated from WT or *Adap*^−/−^ mice were infected or not infected with LCMV-Arm virus for 7 days. The cells were collected and incubated with ConA beads for 10 min at room temperature. The cell-bead complex was incubated with IgG control and ZEB2 antibodies (Proteintech, Cat# 677514-1-Ig) at 4°C overnight. After binding to the pG-MNase enzyme, the cell-beads complex was fragmented using CaCl_2_ solution at 4°C for 2 h. Stop buffer was added to stop fragmentation and DNA was extracted using column-based extraction reagents. The resulting DNA products were quantified by qPCR and spike-in DNA was used as a control. The sequences of the primers for the *Smad3* promoter were as follows: site 1: sense: 5′-GCCTGAGACCATTCCTGGAG-3′, anti-sense: 5′-CCCGGTCATCGTTCTTGGTT-3’; site 2: sense: 5′-CTCTGACCCTTGGTGCTGTG-3′, anti-sense: 5′-GAGACAAGCTCCGGTCGAAA-3’.

#### ELISA

The reagent mixture was equilibrated to room temperature. Add 100 μL of standard working buffer (diluted according to the instructions) or 100 μL of samples to each well and incubate at 37°C for 80 min. Discard the liquid from the plate, add 200 μL of 1 × washing buffer to each well, wash the plate 3 times, and pat the plate dry. Next, 100 μL of 1 × biotinylated antibody working solution was added to each well and incubate at 37°C for 50 min. Discard the liquid from the plate, add 200 μL of 1 × washing buffer to each well, wash the plate 3 times, and pat the plate dry. Then, 100 μL of 1 × streptavidin HRP working solution was added to each well and incubate at 37°C for 50 min. Discard the liquid from the plate, add 200 μL of 1 × washing buffer to each well, wash the plate 5 times, and pat the plate dry. Add 90 μL of TMB substrate solution to each well and incubate at 37°C in the dark for 20 min. Add 50 μL of stop solution to each well, shake the plate for 1 min to mix, the data were recorded at an OD value of 450 nm, and calculate the results.

### Quantification and statistical analysis

#### Statistical analysis

All the streaming data were analyzed using FACS Canto II and FlowJo V10 professional streaming software. GraphPad Prism8 was used to plot and process experimental data. An unpaired Student’s *t* test was used to compare the means of two samples. For groups of more than two groups, one-way ANOVA or two-way ANOVA followed by Tukey’s or Bonferroni’s multiple comparison test was used, depending on the situation. Measurement data are expressed as mean ± standard deviation (SD). n represents the number of independent biological replicates per group, and exact sample sizes are indicated in the corresponding figure legends, *p* represents the significance value, and *p* < 0.05 was considered significant. Ns: not significant, ∗*p* < 0.05, ∗∗*p* < 0.01, ∗∗∗*p* < 0.001, ∗∗∗∗*p* < 0.0001.

## Supplemental information

Document S1. Figures S1–S5, Table S1, and Data S1

Table S2. Mass spectrometry proteomics data, related to STAR methods
