# Supplementary material for: The ADAP-TRIM21-ZEB2 axis regulates CD8+ T cell effector expansion and central memory-like differentiation
Source: iScience. 2026 Aug 1;29(8):117016. doi: 10.1016/j.isci.2026.117016 (PMC13453471; doi:10.1016/j.isci.2026.117016)
Supplement: Supplementary file 1 [file mmc1.pdf]

iScience, Volume *29*

Supplemental information

The ADAP-TRIM21-ZEB2 axis regulates $CD8^+$ T cell effector expansion and central memory-like differentiation

Jiajia Lu, Fan Yang, Yanli Li, Pengchao Zhang, Tengfei Jia, and Hebin Liu

Figure S1

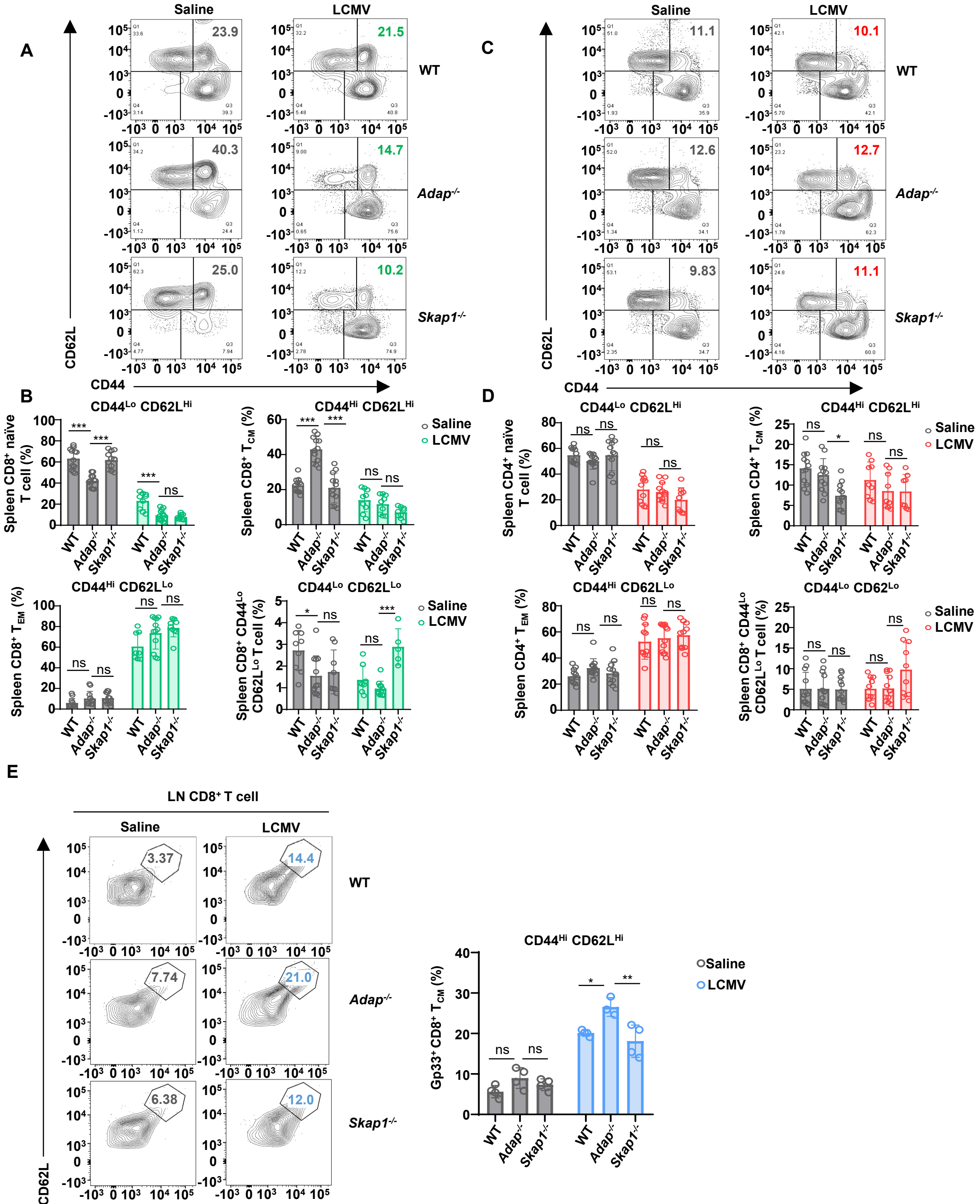

A
B
C
D
E
Spleen CD8⁺ T cell
Spleen CD4⁺ T cell
Saline
LCMV
WT
Adap-/-
Skap1-/-
CD62L
CD44
CD44Lo CD62LHi
CD44Hi CD62LHi
CD44Hi CD62LLo
CD44Lo CD62LLo
Spleen CD8⁺ naïve T cell (%)
Spleen CD8⁺ TCM (%)
Spleen CD8⁺ TEM (%)
Spleen CD8⁺ CD44Lo CD62LLo T cell (%)
Spleen CD4⁺ naïve T cell (%)
Spleen CD4⁺ TCM (%)
Spleen CD4⁺ TEM (%)
LN CD8⁺ T cell
Gp33⁺ CD8⁺ TCM (%)
ns

Figure S1. The loss of ADAP specifically increases the frequency of $CD8^+$ T_{CM}-like cells in the lymph nodes, rather than in the spleen. Related to Figure 3. (A, C) Analysis of the differentiation of splenic $CD4^+$/$CD8^+$ T-cell subsets on day 7 p.i. or in the saline control group. Representative contour plots showing naïve ($CD44^{Lo}$ $CD62L^{Hi}$), T_{CM} ($CD44^{Hi}$ $CD62L^{Hi}$), T_{EM} ($CD44^{Hi}$ $CD62L^{Lo}$) and $CD44^{Lo}$ $CD62L^{Lo}$ cells from WT, *Adap*$^{-/-}$ and *Skap1*$^{-/-}$ mice. The frequencies of the indicated subsets were shown in **(B, D)** (n ≥ 5 mice/group, two-way ANOVA, Bonferroni's multiple comparison). **(E)** Analysis of differentiation of lymph node Gp33-specific $CD8^+$ T_{CM}-like cells ($Gp33^+$ $CD44^{Hi}$ $CD62L^{Hi}$) on day 7 p.i. or treated with saline. Representative contour plots (left) showing Gp33-specific $CD8^+$ T_{CM}-like cells ($Gp33^+$ $CD44^{Hi}$ $CD62L^{Hi}$) from WT, *Adap*$^{-/-}$ and *Skap1*$^{-/-}$ mice. The frequencies of the indicated subsets were shown on right (n ≥ 3 mice/group, two-way ANOVA, Tukey's multiple comparison). All data are expressed as mean with SD. n represents biological replicates per group. **p* < 0.05, ***p* < 0.01, ****p* < 0.001, ns: not significant.

Figure S2

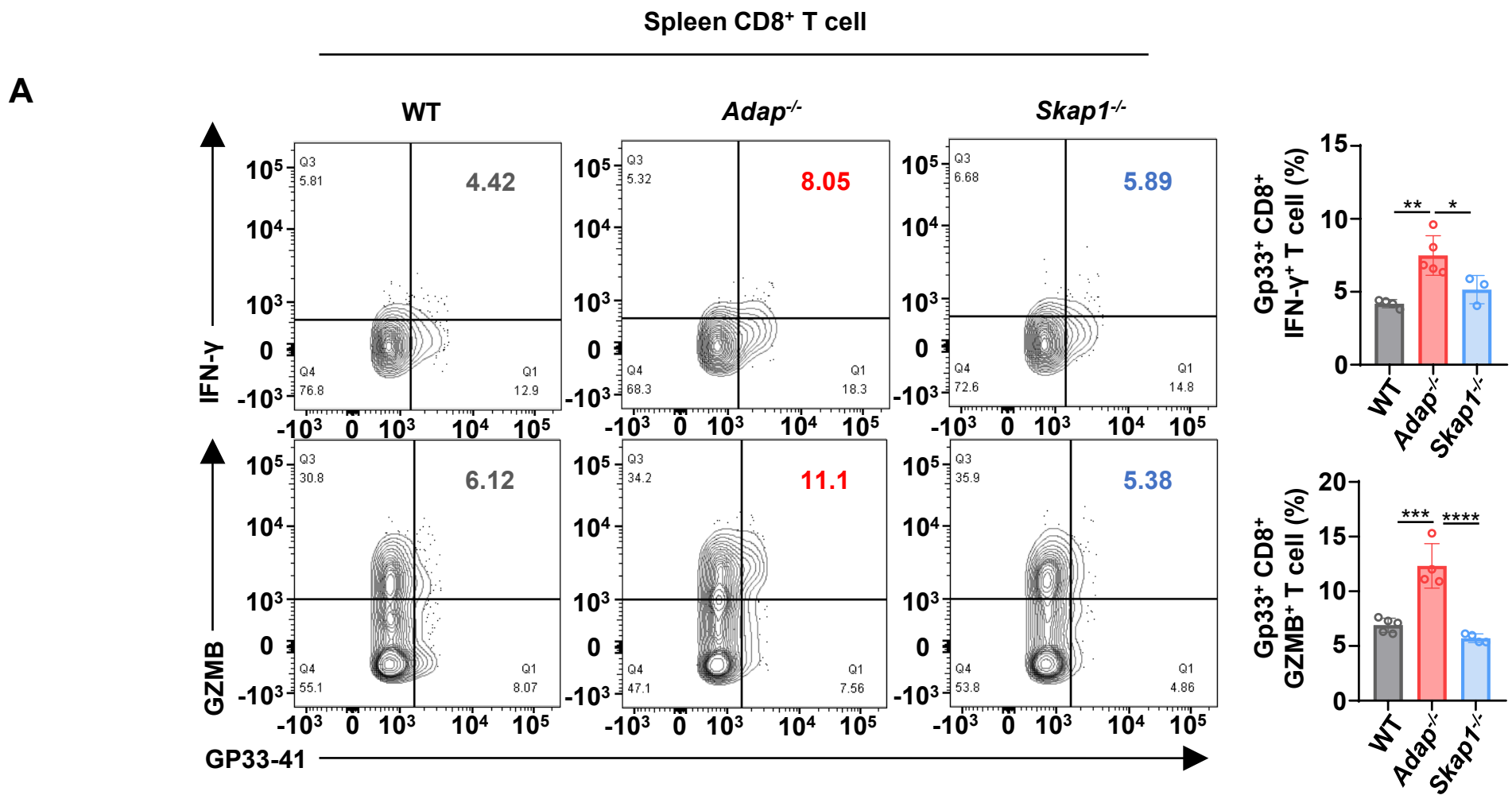

Figure S2. ADAP deficiency promotes antigen-specific CD8⁺ T-cell effector function. Related to Figure 4. (A) Production of cytokines including IFN-γ⁺ and GZMB⁺ cells represented as percentage of Gp33-specific CD8⁺ T cells. Representative contour plots showing the production of IFN-γ and GZMB from WT, *Adap-/-*, and *Skap1-/-* mouse Gp33⁺ CD8⁺ T cells on day 7 p.i.. The frequencies of the indicated subsets were shown on right (n ≥ 3 mice/group, one-way ANOVA, Tukey's multiple comparison). All data are expressed as mean with SD. n represents biological replicates per group. **p* < 0.05, ***p* < 0.01, ****p* < 0.001, *****p* < 0.0001.

Figure S3

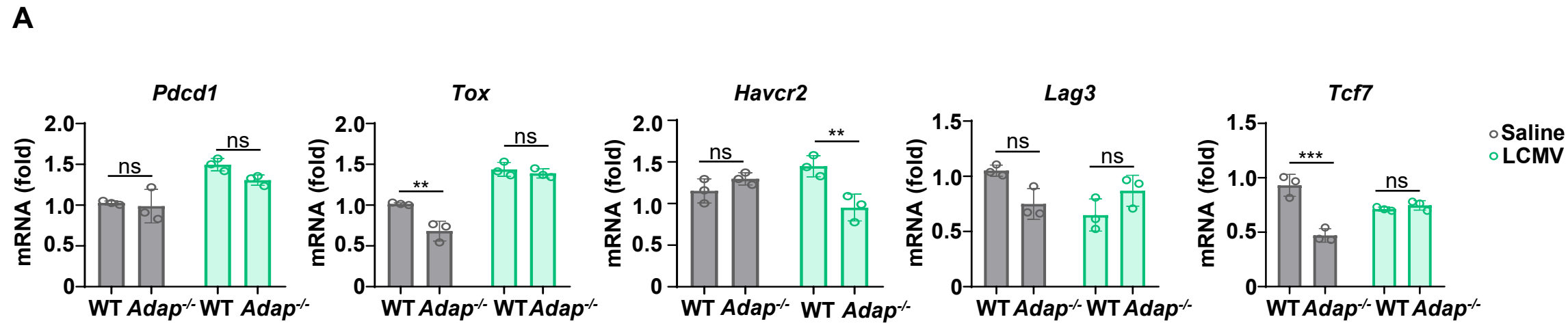

Figure S3. Unchanged expression of exhaustion-related genes in ADAP-deficient CD8+ T cells. Related to Figure 4. (A) Analysis of key regulatory genes expression of exhausted CD8+ T cells. Expression of relative transcripts from WT and *Adap-/-* mice exhausted CD8+ T cells was measured by RT-qPCR on day 30 p.i. or in the saline control group (n = 3 mice/group, two-way ANOVA, Bonferroni's multiple comparison). All data are expressed as mean with SD. n represents biological replicates per group. $^{**}p < 0.01$, $^{***}p < 0.001$, ns: not significant.

Figure S4

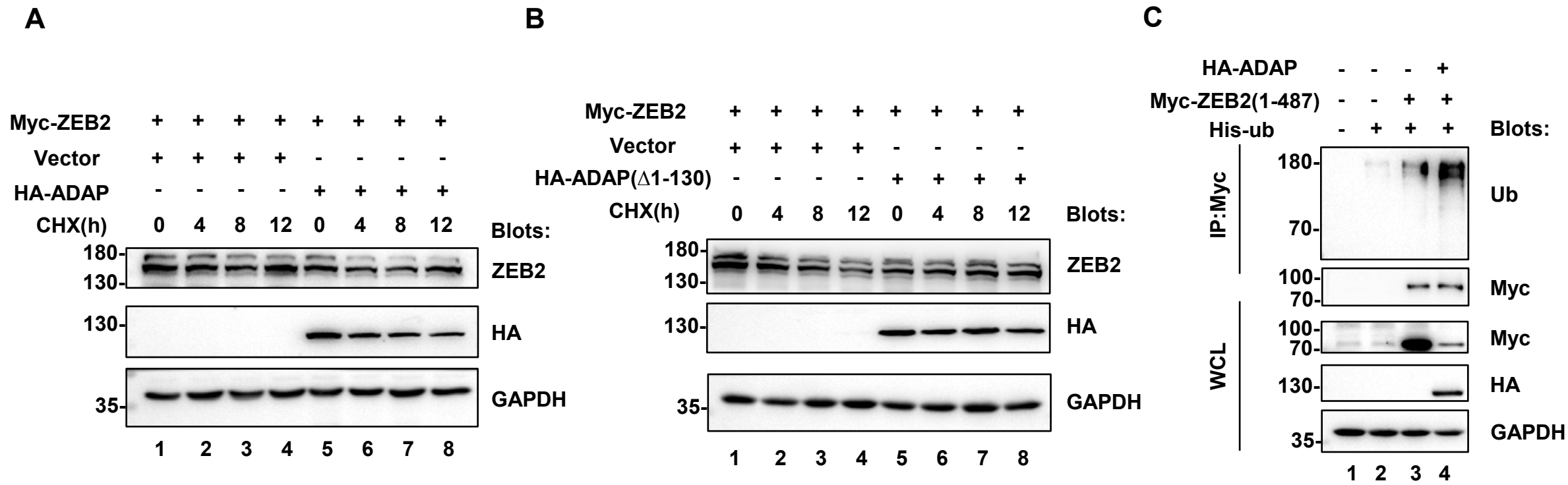

Figure S4. ADAP promotes ubiquitin-mediated ZEB2 degradation. Related to Figure 7. **(A, B)** Expression levels of ZEB2 in HEK293T cells treated with the protein synthesis inhibitor cycloheximide (CHX) for the indicated times (n = 3 independent repeat experiments). **(C)** Co-transfection of HEK293T cells with Myc-ZEB2(1-487), HA-ADAP and His-Ub, 48 h post transfection, ubiquitination of Myc-ZEB2(1-487) was determined by immunoprecipitation with an anti-Myc antibody followed by immunoblotting with the indicated antibodies (n = 3 independent repeat experiments). n represents biological replicates per group.

Figure S5

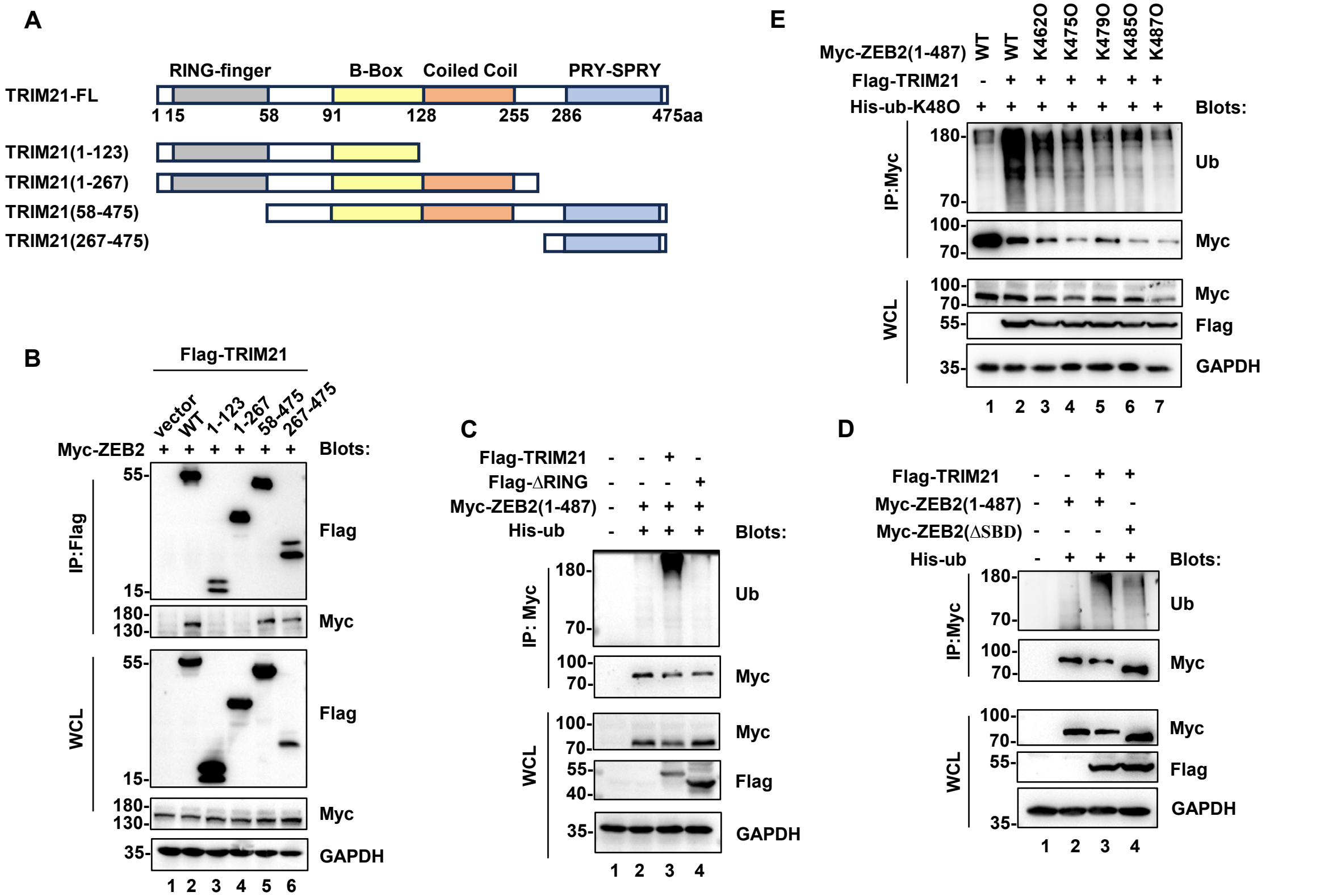

Figure S5. TRIM21 mediates the K48-linked polyubiquitination of ZEB2 at K475. Related to Figure 7. (A) Schematic diagram illustrating the structural domains of TRIM21 and its truncated mutants. **(B)** Myc-ZEB2 was co-expressed with an empty vector, Flag-TRIM21 WT, or Flag-tagged TRIM21 truncation mutants in HEK293T cells. Cell lysates were subjected to immunoprecipitation using an anti-Flag antibody, followed by blotting with anti-Myc and anti-Flag antibodies (n = 3 independent repeat experiments). **(C)** The ubiquitination of Myc-ZEB2(1-487) in HEK293T cells transfected with Flag-TRIM21 (WT) or the Flag-TRIM21 mutant (ΔRING) (n = 3 independent repeat experiments). **(D)** The ubiquitination of Myc-ZEB2(1-487) and its truncation mutant Myc-ZEB2 (ΔSBD) in HEK293T cells transfected with Flag-TRIM21 (n = 3 independent repeat experiments) . **(E)** Ubiquitination site screening of ZEB2 by TRIM21 with indicated site mutants of Myc-ZEB2(1-487) (K462O, K475O, K479O, K485O and K487O) and WT Myc-ZEB2(1-487) in HEK293T cells (n = 2 independent repeat experiments). n represents biological replicates per group.

Table S1. Primers for Quantitative RT-PCR, related to STAR methods

Gene	Forward 5'-3'	Reverse 5'-3'
mZEB2	GCAGTGAGCATCGAAGAGTACC	GACCTTGAGGTCTACGAAAACGG
mGZMA	GTGGTGGAAAGGACTCCTGCAA	GGACCACAGATATGAAAGGAGAG
mGZMB	CAGGAGAAGACCCAGCAAGTCA	GGTTCTCCTGATCTCGACACTC
mIFN-γ	CAGCAACAGCAAGGCGAAAAAGG	TAGGTCGGAGTCCTTCGCCTTT
mGAPDH	CATCACTGCCACCCAGAAGACTG	GACTTGCCCTTCGAGTGACCGTA
mKLRG1	CGAGGAATGGTAGCCACTGTTAC	CCAGTCCTGAAAATCACCTAGCC
mTBX21	CCACCTGTTGTGGTCCAAGTTC	GTTCGGTAATGTCCTACAAACACC
mPRDM1	AAGACGTTCGGTCAGCTCTCCA	TCTCTTCGGTGTACTCACGGTC
mID2	TCACCAGAGACCTGGACAGAAC	GACTCGAATACAGCTTACTATCGT
mPDCD1	CGGTTTCAAGGCATGGTCATTGG	CCTTCGTTCCTGCTGTGAGACT
mTOX	AAGATGGCGCACTGCTCTCCAA	GTAGTCTGTCGTCCGTTCGTAC
mHAVCR2	ACAGACACTGGTGACCCTCCAT	TAACCTCACCCTCAGAGACGAC
mLAG3	CTCCATCACGTACAACCTCAAGG	ACGAGTAACGGTTCACCTGAGG
mTCF7	CCTGCGGATATAGACAGCACTTC	ACCCTAGACCACATGGACCTGT

Data S1. Original uncropped western blot images supporting the main and supplemental figures.

Figure 6

B

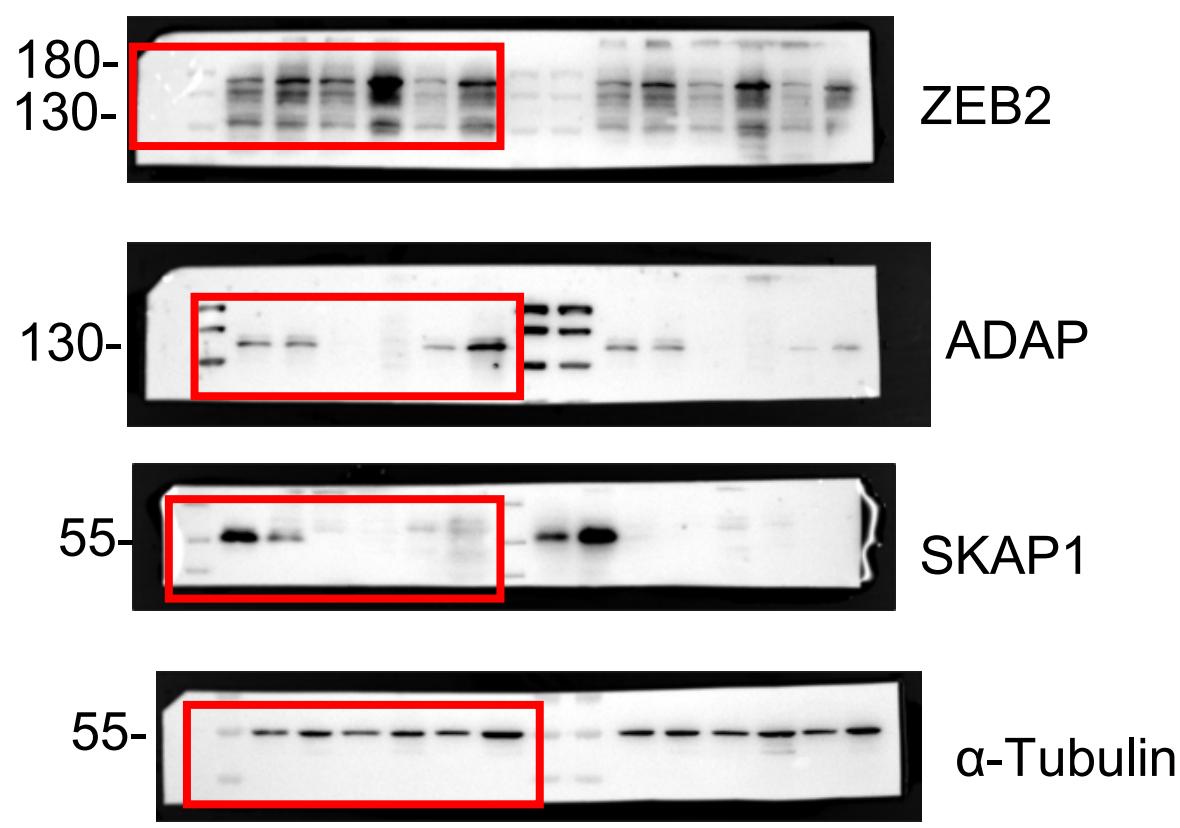

G

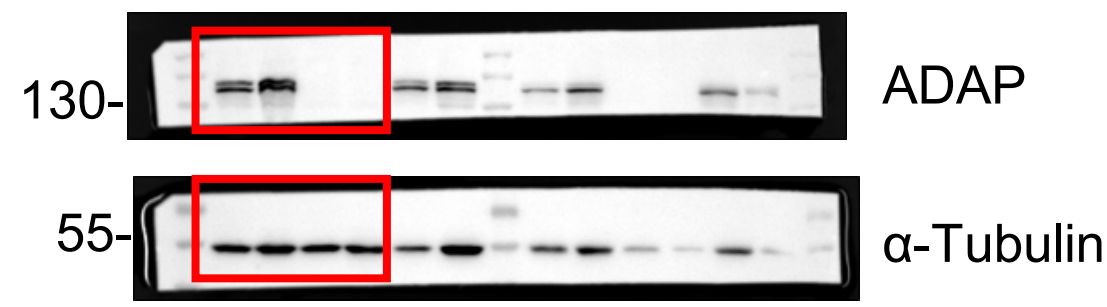

Figure 7

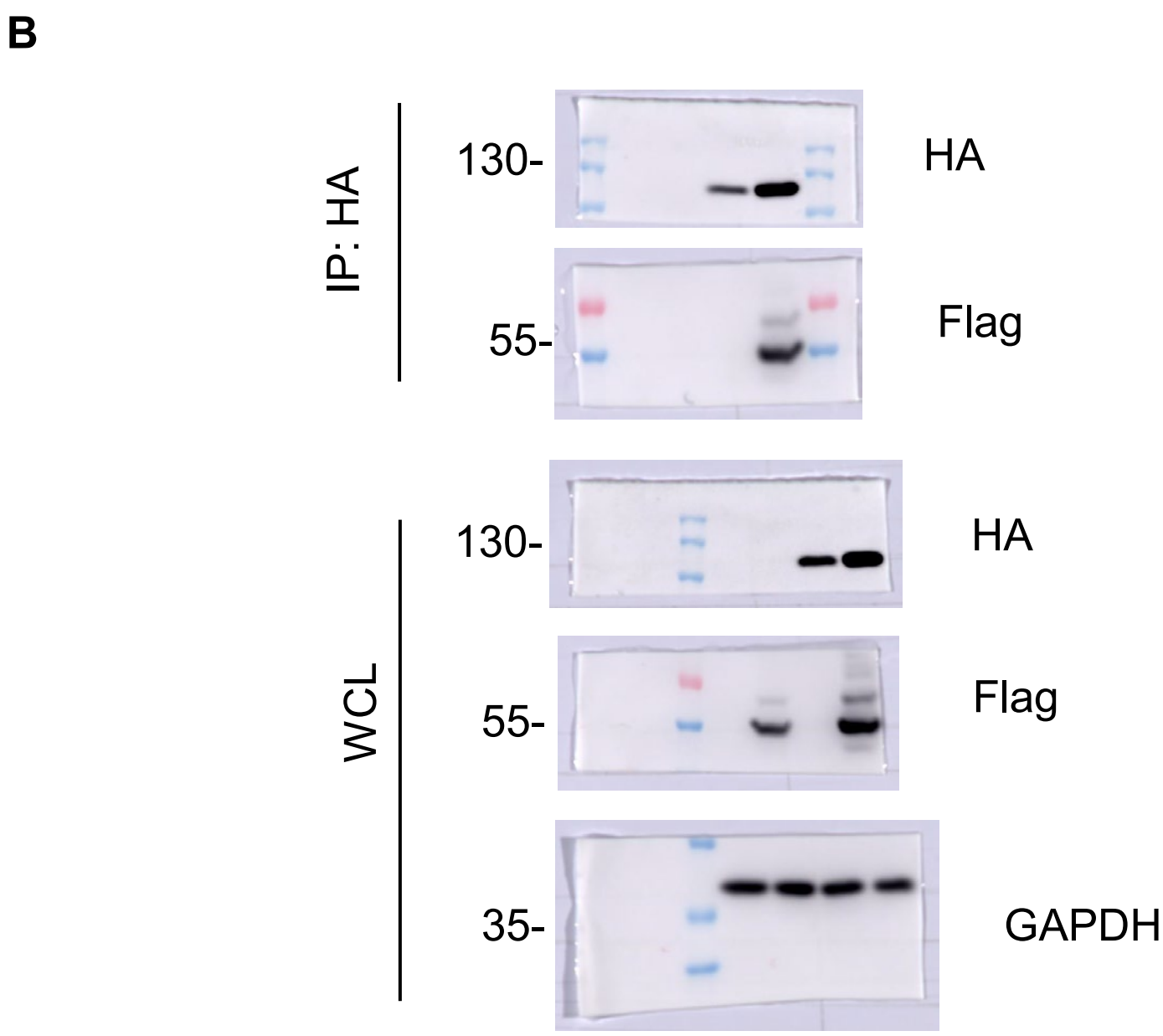

Figure 7

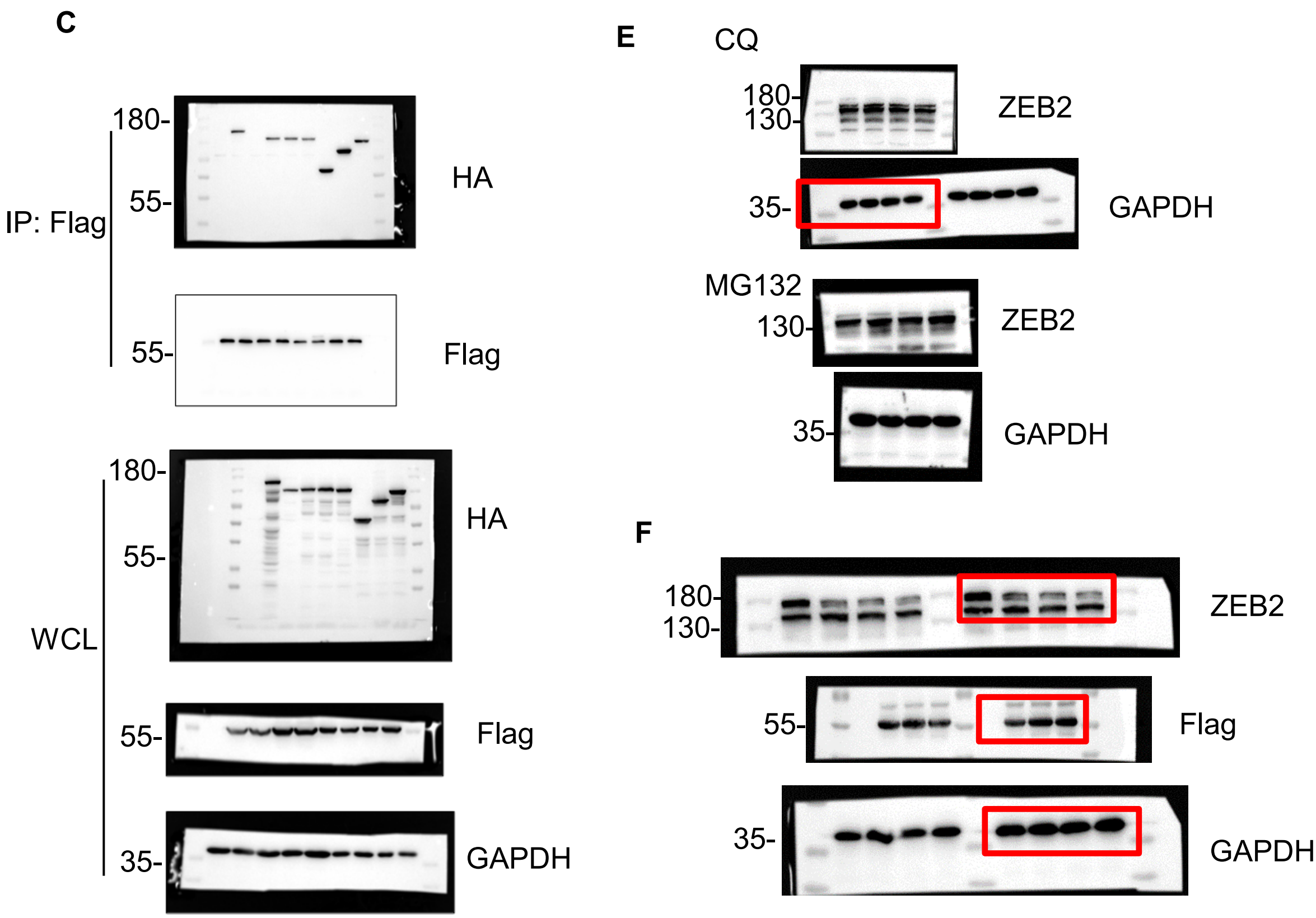

C
180-
55-
HA
IP: Flag
55-
Flag
180-
55-
HA
WCL
55-
Flag
35-
GAPDH
E
CQ
180-
130-
ZEB2
35-
GAPDH
MG132
130-
ZEB2
35-
GAPDH
F
180-
130-
ZEB2
55-
Flag
35-
GAPDH

Figure 7

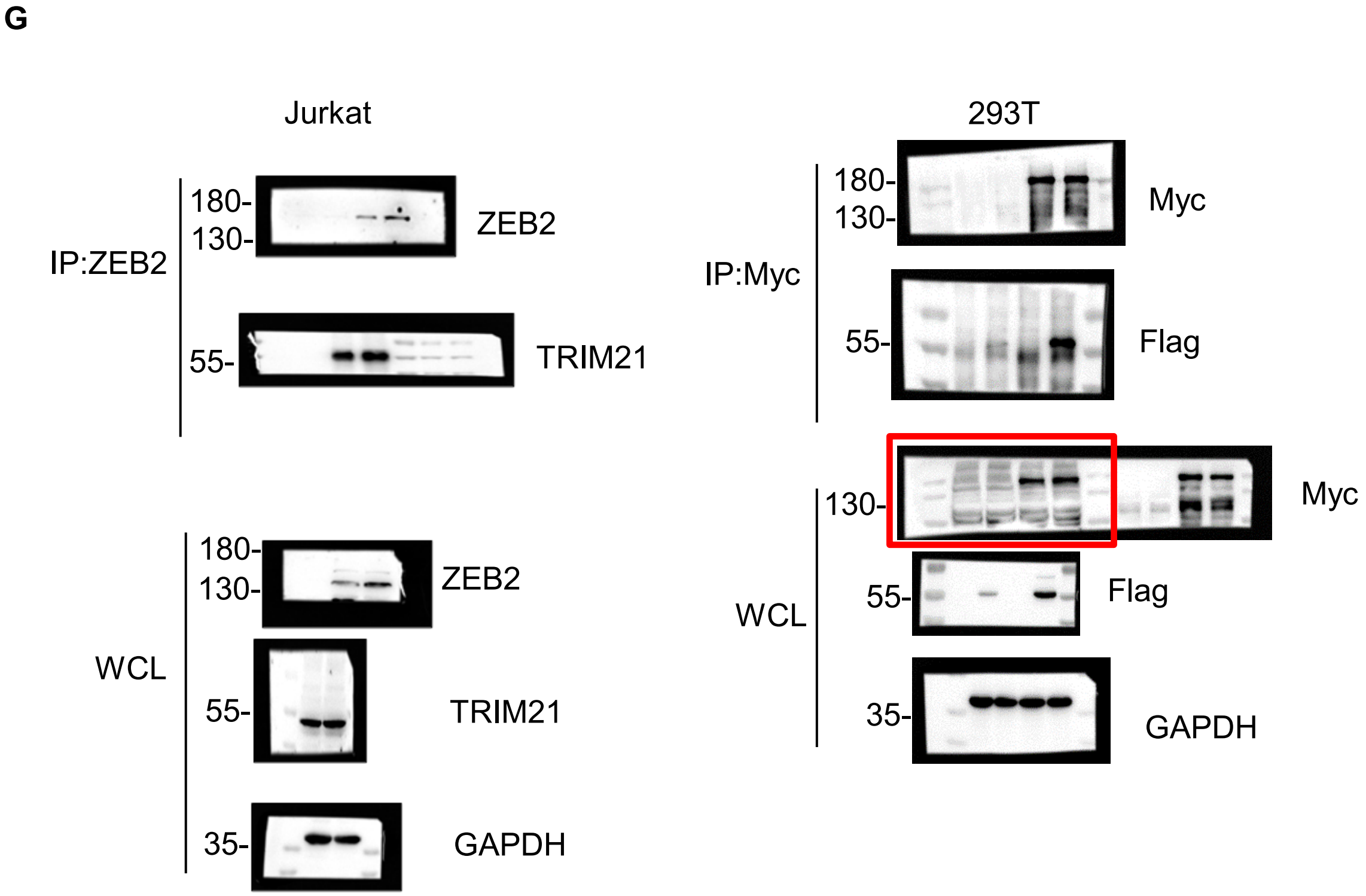
G
Jurkat
IP:ZEB2
180-
130-
ZEB2
55-
TRIM21
WCL
180-
130-
ZEB2
55-
TRIM21
35-
GAPDH
293T
IP:Myc
180-
130-
Myc
55-
Flag
WCL
130-
Myc
55-
Flag
35-
GAPDH

Figure 7

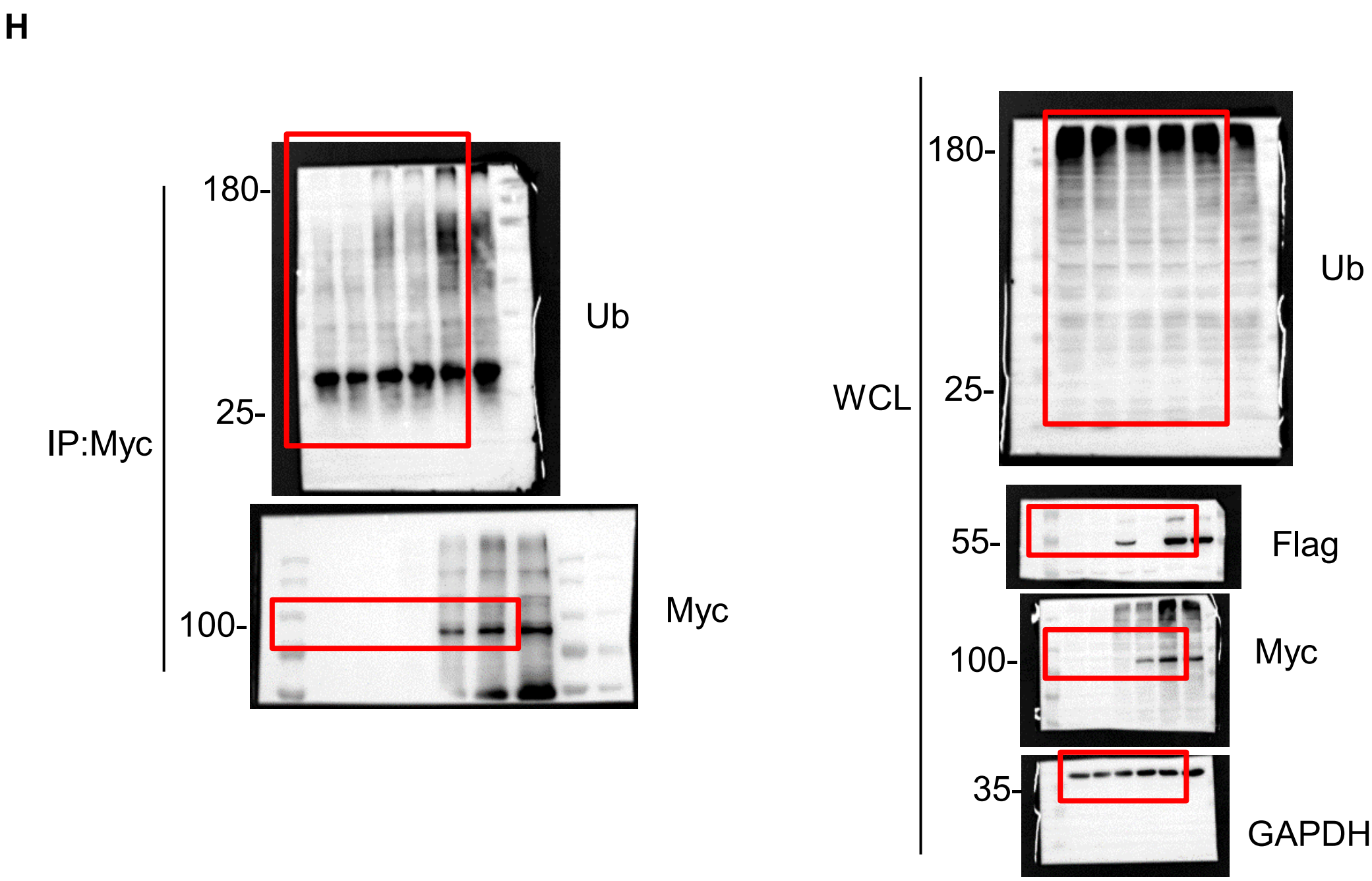

Figure 7

I

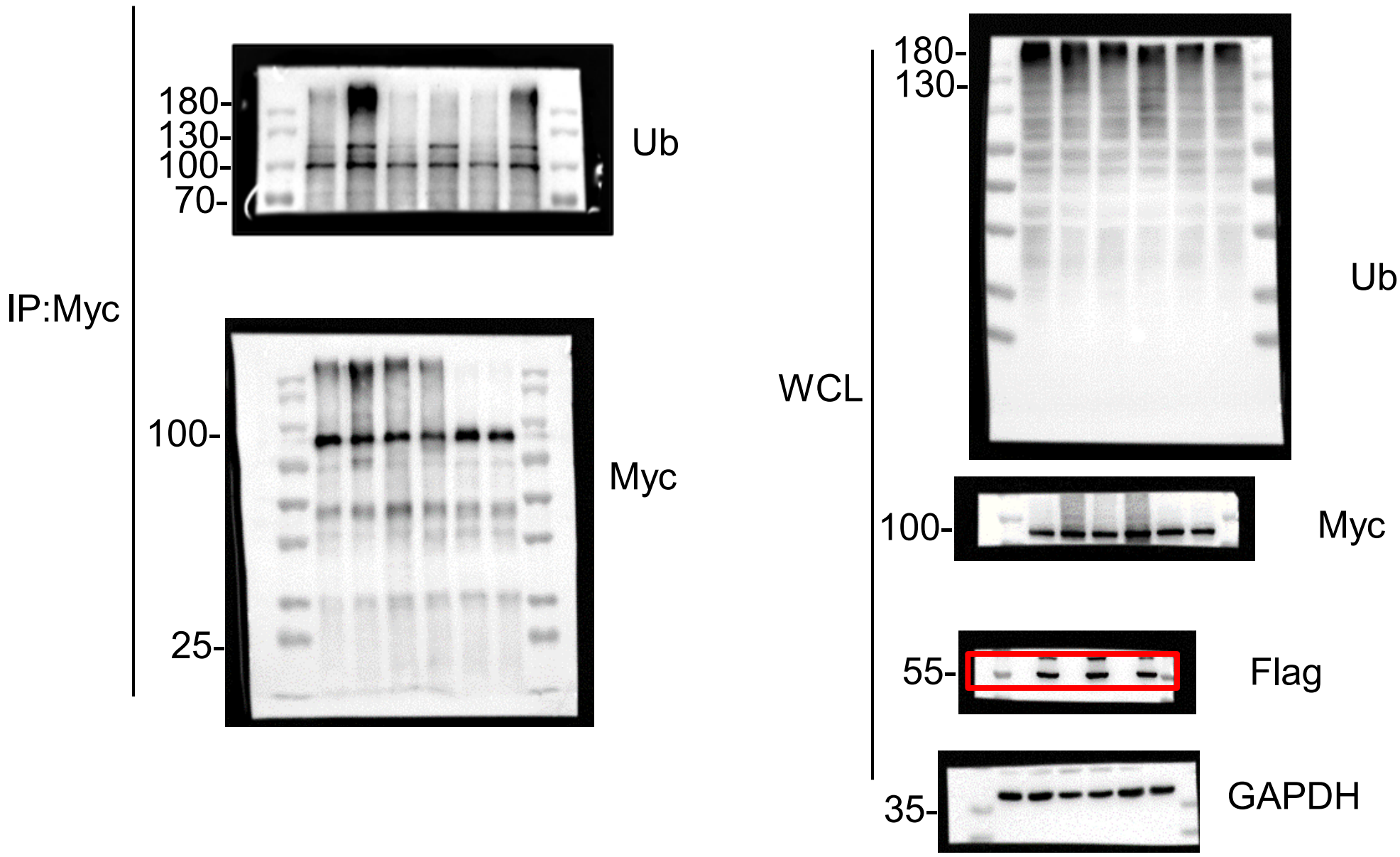

IP:Myc
180-
130-
100-
70-
Ub
100-
25-
Myc
WCL
180-
130-
Ub
100-
Myc
55-
Flag
35-
GAPDH

Figure S4

A

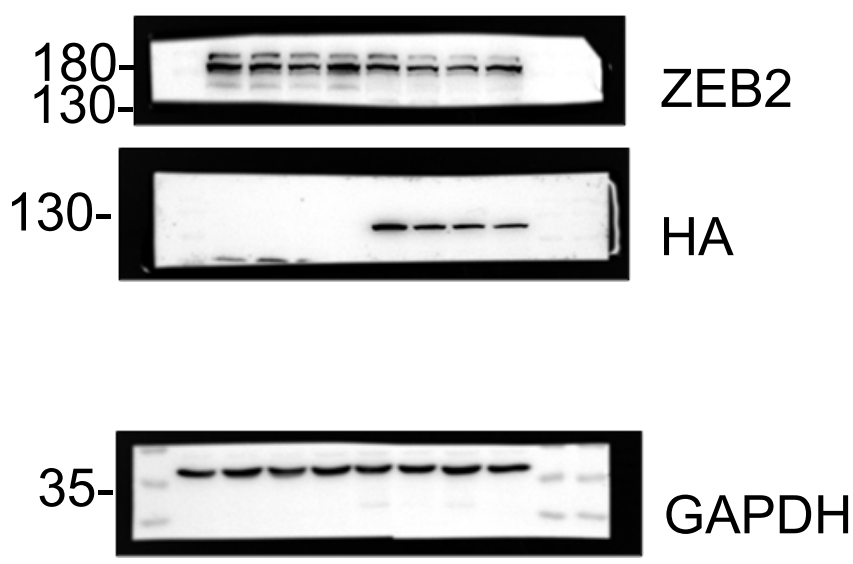

B

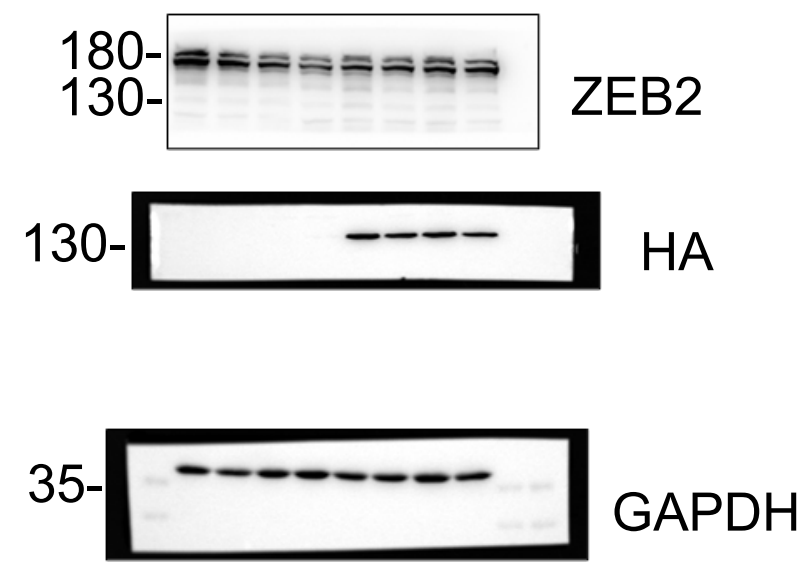

C

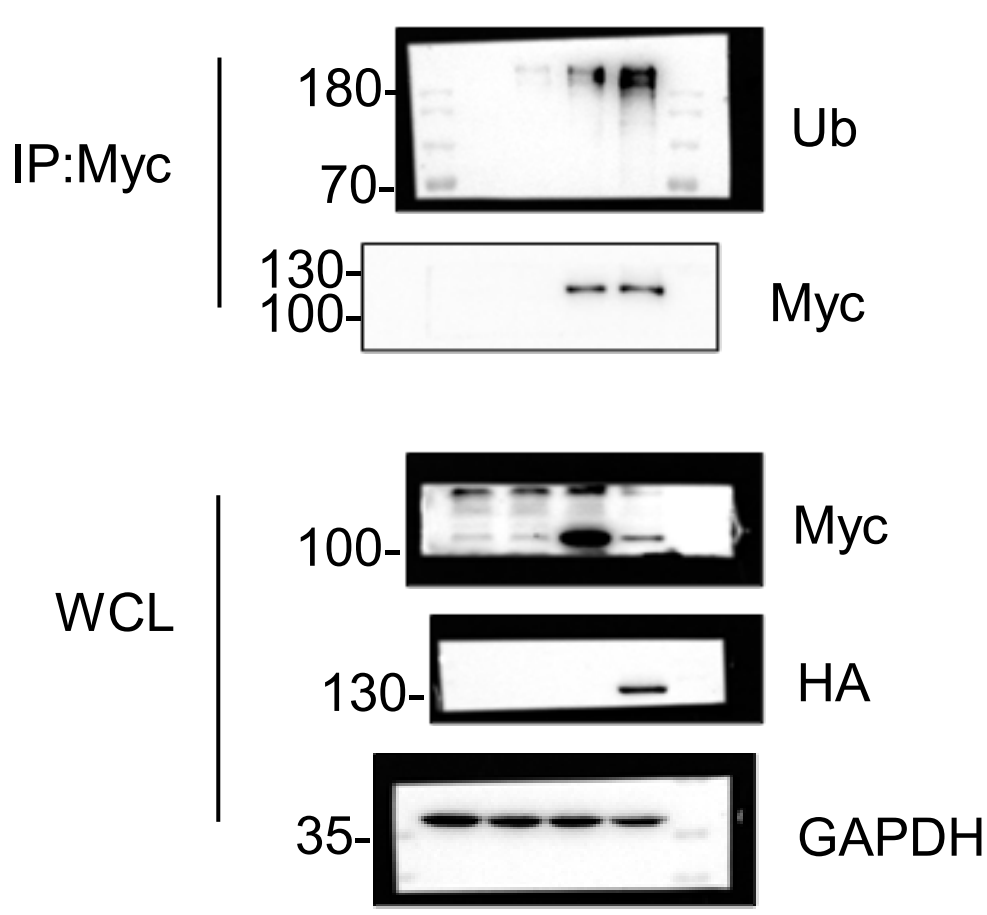

Figure S5

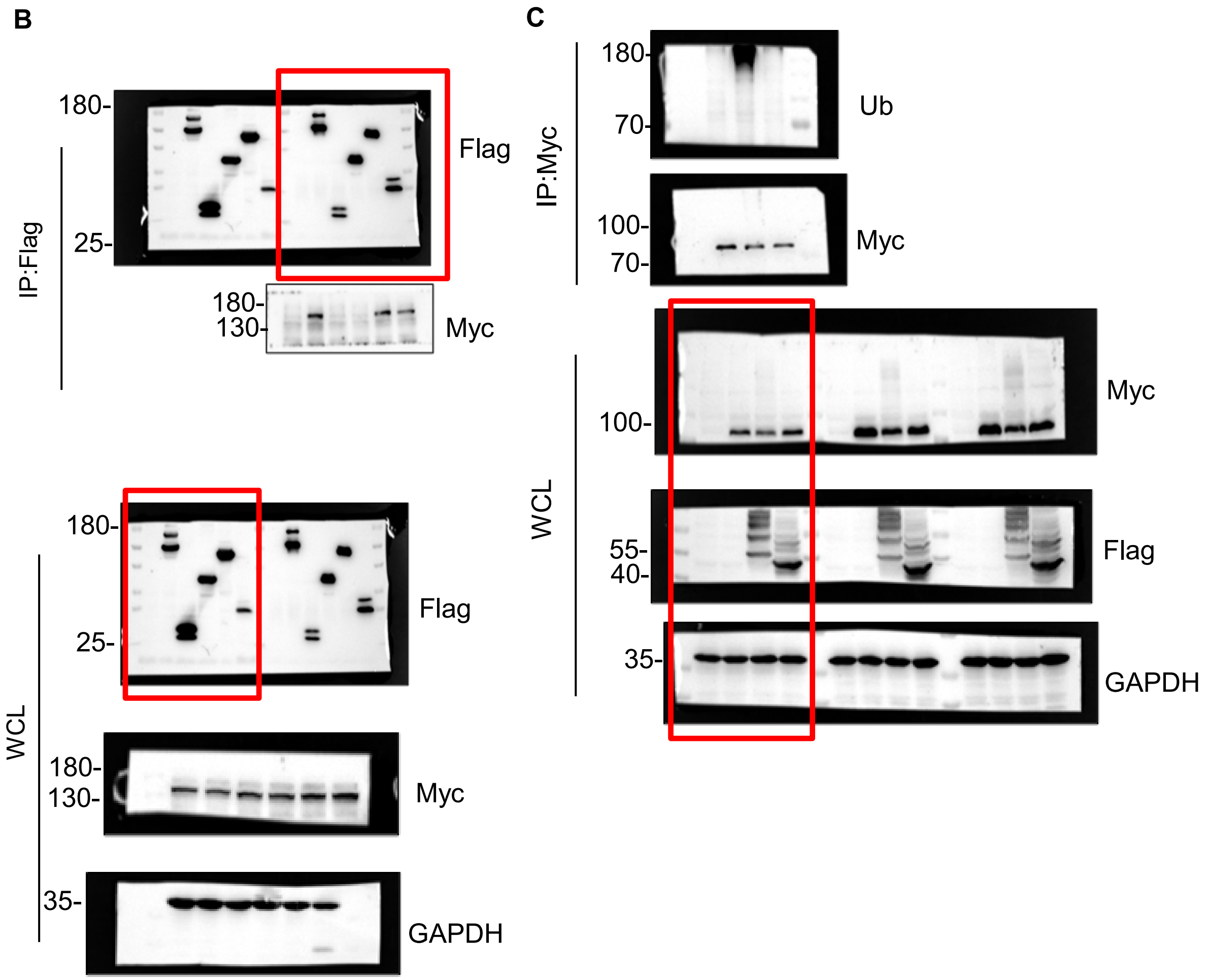
B
180-
25-
Flag
IP:Flag
180-
130-
Myc
WCL
180-
25-
Flag
180-
130-
Myc
35-
GAPDH
C
IP:Myc
180-
70-
Ub
100-
70-
Myc
WCL
100-
Myc
55-
40-
Flag
35-
GAPDH

Figure S5

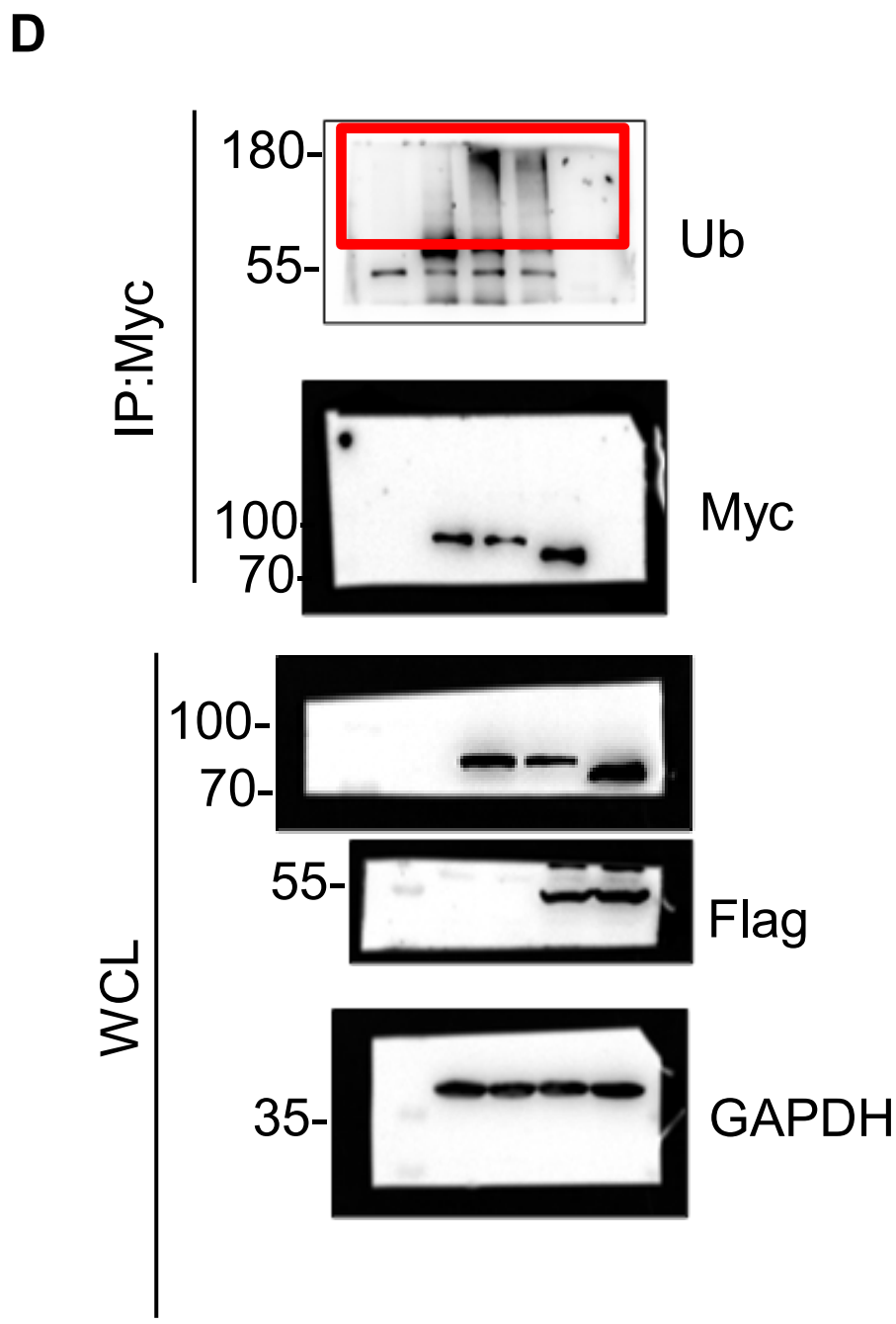
D
IP:Myc
180-
55-
Ub
100
70
Myc
WCL
100-
70-
55-
Flag
35-
GAPDH

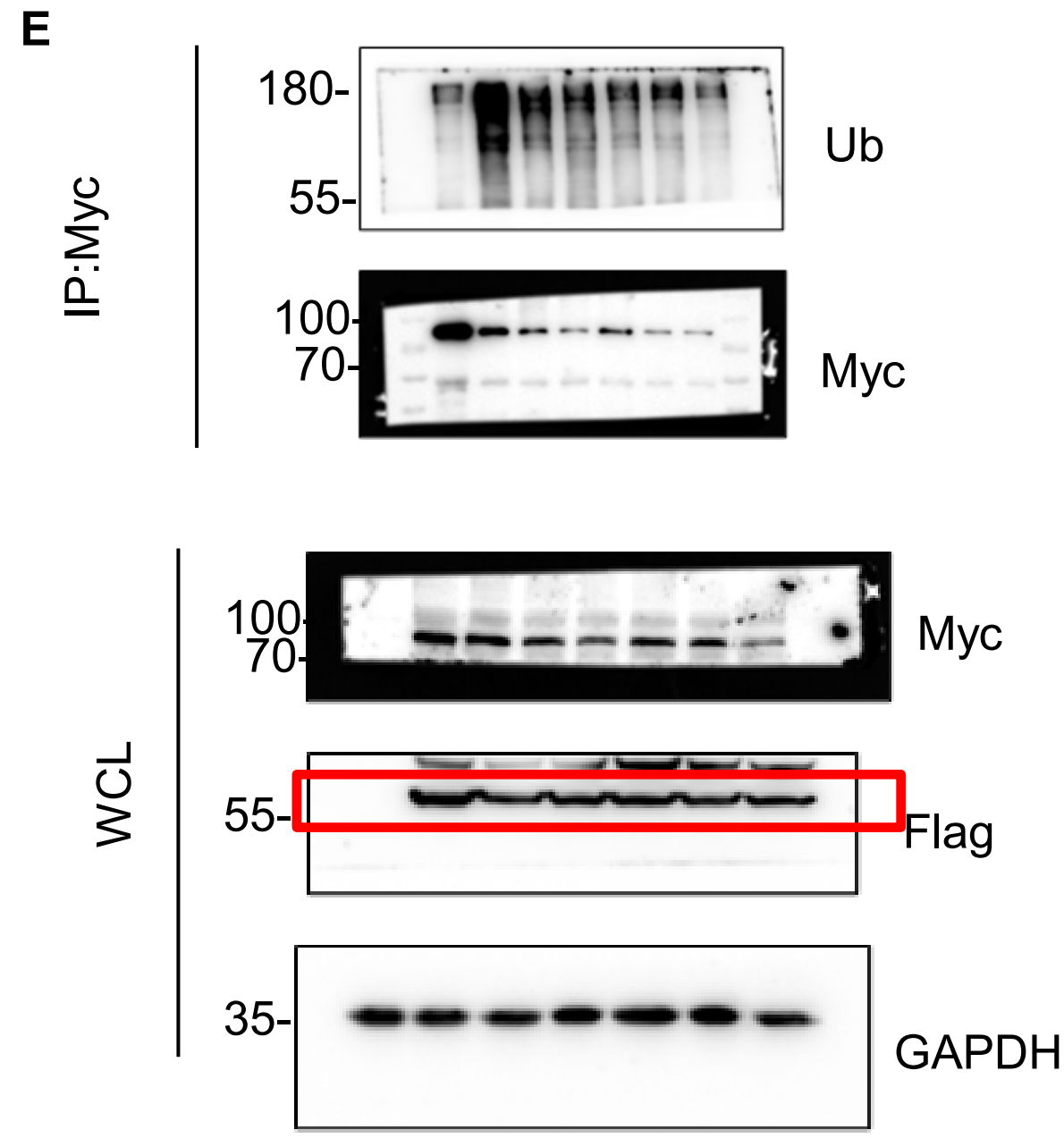
E
IP:Myc
180-
55-
Ub
100
70
Myc
WCL
100
70
Myc
55-
Flag
35-
GAPDH
